# Chemical priming of hypoxia responses via PLANT CYSTEINE OXIDASE inhibition improves flooding tolerance

**DOI:** 10.1093/plphys/kiag505

**Published:** 2026-07-14

**Authors:** Francesco Fioriti, Rocco Pierpaolo Germano, Noemi La Monaca, Dona M Gunawardana, Rebecca Latter, Antonietta Santaniello, Emily Flashman, Paolo Maria Triozzi, Pierdomenico Perata

**Affiliations:** Institute of Plant Sciences, Sant’Anna School of Advanced Studies, Via Guidiccioni 10, San Giuliano Terme, Pisa 56010, Italy; Institute of Plant Sciences, Sant’Anna School of Advanced Studies, Via Guidiccioni 10, San Giuliano Terme, Pisa 56010, Italy; Institute of Plant Sciences, Sant’Anna School of Advanced Studies, Via Guidiccioni 10, San Giuliano Terme, Pisa 56010, Italy; Department of Biology, University of Oxford, South Parks Road, OX1 3EL Oxford, United Kingdom; Department of Chemistry, University of Oxford, Mansfield Road, OX1 3TA Oxford, United Kingdom; Department of Chemistry, University of Oxford, Mansfield Road, OX1 3TA Oxford, United Kingdom; Syngenta Biologicals (Valagro SpA), Via Cagliari 1, Atessa, Chieti 66041, Italy; Department of Biology, University of Oxford, South Parks Road, OX1 3EL Oxford, United Kingdom; Institute of Plant Sciences, Sant’Anna School of Advanced Studies, Via Guidiccioni 10, San Giuliano Terme, Pisa 56010, Italy; Institute of Plant Sciences, Sant’Anna School of Advanced Studies, Via Guidiccioni 10, San Giuliano Terme, Pisa 56010, Italy

## Abstract

Extreme flooding driven by climate change demands strategies to enhance plant tolerance to low-oxygen stress. We investigated the ability of bioactive compounds to chemically prime plants for flooding events. A high-throughput chemical genetic screen of 2,237 bioactive molecules was performed using an *Arabidopsis thaliana* luciferase-based reporter line driven by the *ALCOHOL DEHYDROGENASE* promoter, a hypoxia-inducible gene involved in anaerobic metabolism. The screen identified chlorquinaldol (CQD), an 8-hydroxyquinoline derivative, as a potent inducer of hypoxia responses. CQD inhibits the activity of PLANT CYSTEINE OXIDASES (PCOs), which are crucial oxygen-sensing enzymes in plants, resulting in the stabilization of the ETHYLENE RESPONSE FACTOR type VII and activation of hypoxia-responsive transcription under normoxic conditions. Plants pretreated with CQD showed enhanced tolerance to waterlogging and submergence, indicating that chemical priming of hypoxia responses improves plant tolerance to flooding. This study demonstrates that chemical inhibition of PCOs is an effective strategy for priming hypoxia responses and improving flooding tolerance in plants.

## Introduction

Climate change is driving extreme weather events with significant economic consequences, including reductions in crop yields (Newman and Noy 2023). One of the significant issues is the occurrence of intense and frequent precipitation, which leads to severe flooding and considerable agricultural losses, primarily due to waterlogging or plant submersion (Loreti et al. 2016). Many land plants, including major crops, are susceptible to waterlogging and submergence because gas diffusion decreases, hindering both cellular respiration and photosynthesis (Bailey-Serres and Voesenek 2008). In these hypoxic conditions, plants shift to hypoxic metabolism to maintain energy production, which includes the activation of ethanol and lactate fermentation pathways (Van Veen et al. 2024). This metabolic adjustment, which requires the transcriptional activation of the core hypoxia-responsive genes (HRGs) (Mustroph et al. 2009), is orchestrated by a group of five transcription factors belonging to the family of ETHYLENE RESPONSE FACTOR type VII, which are the main transducers of hypoxia responses in land plants (Gibbs et al. 2011; Licausi et al. 2011; Dalle Carbonare et al. 2025). Under aerobic conditions, ERFVIIs are constitutively marked for proteasomal degradation through the PLANT CYSTEINE OXYDASE (PCO) branch of PROTEOLYSIS 6 (PRT6) N-degron pathway (Weits et al. 2014), thus maintaining an off-state of hypoxia responses (Renziehausen et al. 2025). PCO enzymes act as O_2_ sensors, as their activity correlates with O_2_ levels (White et al. 2018). When O_2_ is available, the PCOs catalyze the dioxygenation of the second cysteine residue to cysteine sulfinic acid at the N-termini of ERFVIIs, which in turn are arginylated by arginyl transferases (ATEs) (White et al. 2017). These modifications ultimately render these proteins substrates for the E3 ubiquitin ligase PRT6, which target them for proteasomal degradation (Zubrycka et al. 2023). Under hypoxia, ERFVIIs are stable and translocate to the nucleus where their activity is modulated by additional post-translational modifications that determine the magnitude of hypoxia response activation (Fan et al. 2023; Kunkowska et al. 2023). Thus, the activation of ERFVII-dependent transcriptional responses, including transcripts coding for fermentative enzymes such as *PYRUVATE DECARBOXYLASE* (*PDC*) and *ALCOHOL DEHYDROGENASE* (*ADH*), is crucial for the survival of plants under limited-oxygen conditions such as waterlogging or submergence.

Since ERFVII stabilization is the molecular switch that activates anaerobic metabolism, strategies to confer flooding tolerance could focus on modulating PCO activity (White and Flashman 2016). Substitutions of crucial residues at the active site of the PCO4 enzyme from *Arabidopsis thaliana* generated PCO inactive isoforms and constitutive activation of anaerobic responses (White et al. 2020). Furthermore, recent studies have shown that modifications of key amino acids in the catalytic site of PCOs, or replacing PCO with diverse N-terminal cysteine oxidases, could improve flooding tolerance in Arabidopsis (Dirr et al. 2025; Perri et al. 2025). In addition to genetic engineering of PCO enzymes, PCO activity can be modulated by chemicals that compete with their substrates, covalently bind to these enzymes to modify their activity, or through compounds that chelate the active site Fe(II) (White and Flashman 2016). Indeed, chelating agents such as dipyridyl inhibits the human thiol 2-aminoethanethiol dioxygenase (ADO) (Masson et al. 2019) and also stabilize ERFVIIs likely through inhibition of PCO in Arabidopsis (Telara et al. 2026). Although the low specificity of chelating agents may cause side physiological effects due to nonspecific inhibition of Fe-dependent pathways, a recent study identified hydralazine, a drug used for hypertensive crisis treatment due to its potent vasodilatory activity, as a selective and effective inhibitor of ADO via both chelation of the iron cofactor and alkylation of a coordinating ligand residue (Shishikura et al. 2025). The use of hydralazine as ADO inhibitor demonstrates that some chelating agents can be potent and specific inhibitors of thiol dioxygenase activities.

A recent high-throughput chemical screen using a synthetic yeast platform identified novel small molecules that function as PCO inhibitors (Lavilla-Puerta et al. 2023). Among these hypoxia-mimicking molecules, a substituted bis-cyclohexanedione compound was the most effective PCO inhibitor, likely by competing with substrate for binding at the active site of PCOs (Lavilla-Puerta et al. 2023). Pretreatment using these compounds improved the chlorophyll content in Arabidopsis seedlings treated with anoxia, suggesting that these PCO inhibitors may be involved in chemical priming of hypoxic responses (Lavilla-Puerta et al. 2023). Therefore, identification of chemical compounds that modulate PCO activity and thus hypoxia responses could be of high agronomic value, inducing a priming state that could enhance plant tolerance to subsequent hypoxic stresses.

In plants, strategies for identifying small molecules of interest have relied on chemical genetic screens using reporter systems (Zhao et al. 2003; Armstrong et al. 2004; Yoneda et al. 2007; Schreiber et al. 2008; Kim et al. 2010, 2011; Serrano et al. 2010; Tóth et al. 2012; Motte et al. 2013). Previous works took advantage of the β-glucuronidase (GUS) reporter system for large-scale chemical screens in plants, which requires extensive protocols to reveal GUS activity (Hayashi et al. 2003; Armstrong et al. 2004; Gendron et al. 2008; Knoth et al. 2009; Serrano et al. 2010; Halder and Kombrink 2015). Alternatively, luciferase (LUC) reporter systems have been successfully used for chemical screens in plants, enabling quantitative in vivo imaging of reporter gene activity (Meesters and Kombrink 2014; García-Maquilón et al. 2021; Li et al. 2022).

In this study, we performed a high-throughput chemical genetic screen to test 2237 bioactive molecules of animal, plant, and bacterial sources to test their ability to induce activity of a previously characterized LUC-based transcriptional reporter driven by the *ADH* promoter (Brunello et al. 2024), which served as a proxy for hypoxia response activation. This approach led to the identification of chlorquinaldol (CQD), an 8-hydroxyquinoline derivative, as a potent chemical inducer of hypoxia responses that inhibits PCO activity and promotes plant tolerance to waterlogging and submergence. This work highlights that chemical modulation of PCO activity is a promising strategy to enhance plant survival under oxygen-limiting conditions.

## Materials and methods

### Plant material and growth conditions

The *A. thaliana* Columbia-0 ecotype (Col-0) was used as the wild-type background in all experiments. The pentuple mutant *erfVII* (N2111601) (Abbas et al. 2015), the *adh1* (N552699) mutant and the double mutant *pdc1pdc2* mutant (N660027 crossed with N862662 described by Bui et al. 2019) were obtained from the Nottingham Arabidopsis Stock Centre (NASC). Arabidopsis transgenic lines *35S::RAP2.3^3xHA^* (Gibbs et al. 2014), *pADH::LUC* and *p5xHRPE::LUC* (Brunello et al. 2024) used in this study were previously characterized. For *in vitro* experiments, *A. thaliana* Col-0 seeds were surface-sterilized by incubating them 2 min in 70% (v/v) ethanol, 5 min in 5% (v/v) hypochlorite solution and then washed several times with sterile water. Seeds were stratified at 4 °C in the dark for 48 h and then germinated on square petri dishes (120 × 120 × 17 mm LxWxH, Greiner Bio-One) containing 25 mL of solid MS medium (Murashige and Skoog half-strength medium, 0.5% w/v sucrose, 0.7% phytagel, pH 5.7) under constant temperature of 22 °C, with a photoperiod of 12 h/12 h light/dark cycles and light intensity of 150 μmol m^−2^ s^−1^ photosynthetic photon flux density (PPFD). For soil-grown plants, seeds were stratified as described above and germinated at 22 °C day/20 °C night and with a photoperiod of 12 h/12 h light/dark cycles and light intensity of 150 μmol m^−2^ s^−1^ PPFD. Five-day-old seedlings were transplanted directly on a 3:1 peat/perlite mixture and kept growing in the same conditions.

### Construct preparation and transgenic line generation

*bHLH38* promoter (*pbHLH38*, 1.000 bp upstream of the start codon including the 5′ untranslated region) was amplified from *A. thaliana* Col-0 genomic DNA using Phusion High-Fidelity DNA Polymerase (New England Biolabs). Restriction sites required for GreenGate cloning were added to the primers (Table S4), which was subsequently cloned into pCR2.1 vector (TA Cloning™ Kit, Thermo fisher scientific). GreenGate Cloning System method (Lampropoulos et al. 2013) was used to assemble *pbHLH38* with the fusion protein composed by three times repeat of green fluorescent protein (*3xGFP*; from pGGC025) in frame with the nano luciferase (nLUC; synthetized by Synbio Technologies) and *UBQ10* terminator (*tUBQ10*; from pGGE009) into the final transcriptional unit *pbHLH38::3xGFP-nLUC::tUBQ10* in a single restriction-ligation reaction with T4 DNA ligase (Thermo-Fisher Scientific) and FastDigest *Eco31I* (Thermo-Fisher Scientific) following GreenGate reaction (Lampropoulos et al. 2013). This construct was assembled with the BastaR cassette (pGGF002) into the destination vector (pGGZ001) for plant transformation. The destination vector carrying both *pbHLH38::3xGFP-nLUC::tUBQ10* and *p35S::BastaR::t35S* was transformed into *Agrobacterium tumefaciens* strain GV3101. *Arabidopsis thaliana* Columbia-0 (Col-0) was transformed with Agrobacterium following the floral dipping method (Clough and Bent 1998). Selection of transgenic plants was carried out on medium containing 25 μg/mL of ammonium glufosinate. Homozygous plants were obtained before performing the experiments.

### Chemical and hypoxic treatments

**Figure 1:**
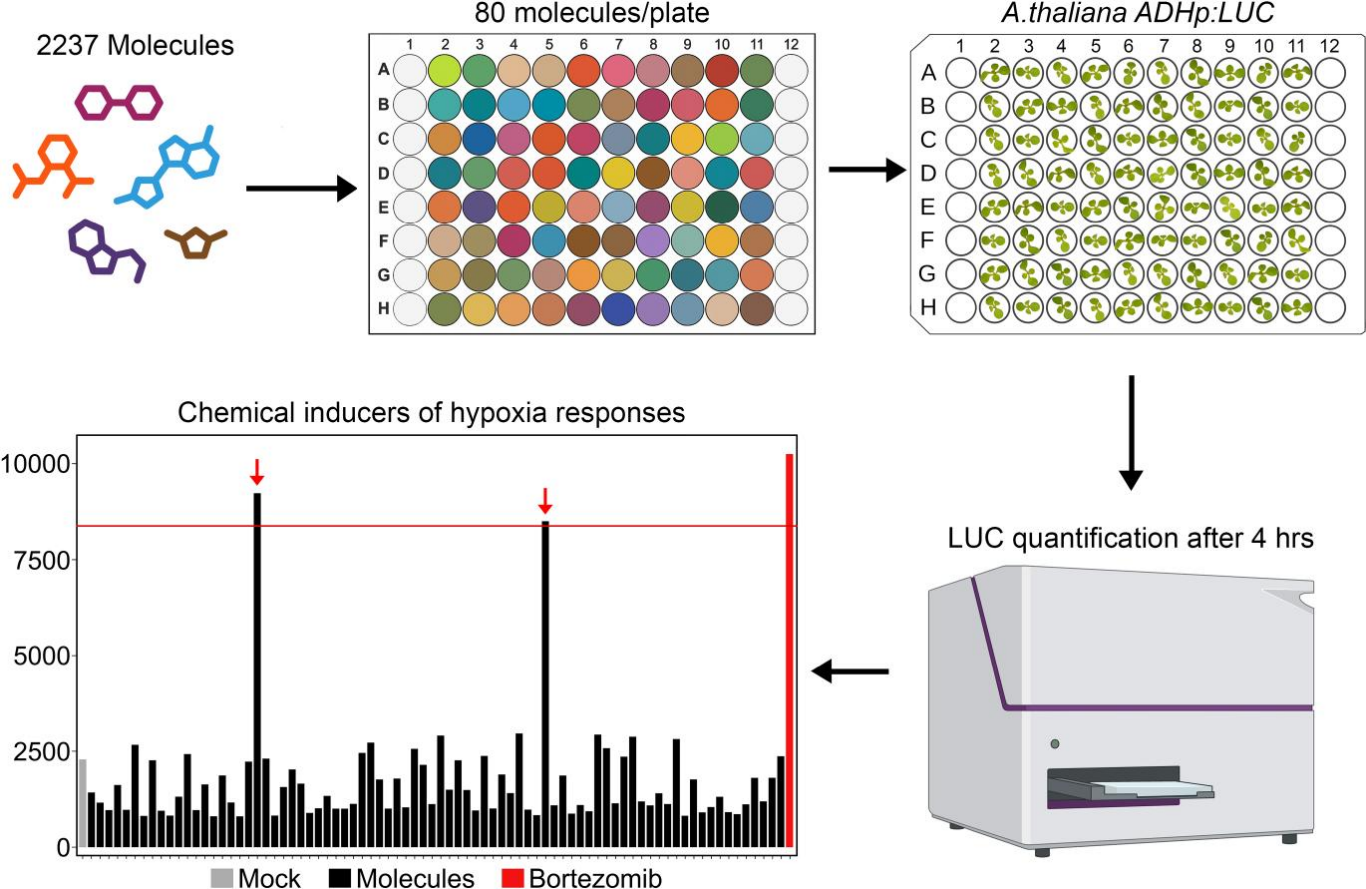
Schematic illustration showing high-throughput chemical genetic screen performed on Arabidopsis *pADH::LUC* reporter line. A chemical library (MedChemExpress) of 2237 molecules was tested on *pADH::LUC* transgenic line. Each molecule of the library was added to each well and luminescence from *pADH::LUC* reporter recorded after 4 h using a multimode plate reader. Horizontal red line indicates the threshold value established based on the luminescence signal emitted by *pADH::LUC* treated with bortezomib (positive control). Arrows depict molecules that induced *pADH::LUC* reporter with a value of luminescence above the established threshold (horizontal line) and thus were selected for further validation. Icons representing the molecules and luminometer were created in BioRender. https://BioRender.com/pv174dt.

For high-throughput chemical screen, a unique collection of natural compunds (Natural Product Library, Cat. No.: HY-L021; MedChemExpress) was obtained with the aim to identify chemical inducers of hypoxia responses in *A. thaliana*. Each molecule was resuspended in dimethyl sulfoxide (DMSO) to a stock concentration of 10 mM. A final working concentration of 100 µM for each molecule was obtained in water. *pADH::LUC* 8-day-old Arabidopsis seedlings grown in 96-well white plates (Thermo-Fisher Scientific) were treated by adding 30 μL of 100 µM of each molecule into each well (Fig. 1). As a positive control treatment, 30 μL of 100 µM of proteosome inhibitor bortezomib was used, as it was previously showed to stabilize ERFVIIs and activate hypoxia responses in Arabidopsis (Zubrycka et al. 2023). The negative control treatment was a solution of water with an equivalent volume of DMSO used to preapre the working concentrations of each molecule. The plates were then sealed, placed back in the growth chamber, and luminescence recorded after 4 h.

To compare the abundance of ADH and PDC proteins after CQD and hypoxic conditions, 8-day-old Arabidopsis seedlings were transferred to an anaerobic workstation (O_2_ Control InVitro Glove Box; Coy Laboratory Products) by flushing an oxygen-modified atmosphere for 4 h (1% [v/v] O_2_/N_2_).

For gene expression and protein abundance analysis, 8-day-old Arabidopsis seedlings growing vertically on square plates were transferred to a square plate containing a medium supplemented with 100 µM of the corresponding molecule, placed back in the growth chamber (zeitgeber time 4, ZT4), and harvested after 4 h (ZT8). The control treatment was prepared by adding the corresponding volume of DMSO to the medium.

For the plant growth assay, 21-day-old Arabidopsis plants were sprayed with either MOCK or 100 µM of CQD solution and HUE and leaf area were recorded every 2 d over a period of 10 d under day/night cycles.

For the root growth assay, 6-day-old Arabidopsis seedlings were transferred to square petri dishes containing solid MS medium supplemented with either MOCK or 100 µM of CQD. After 4 h, Arabidopsis seedlings were transferred to square petri dishes containing solid MS medium, prepared as described above, and root length and lateral root number were recorded every 2 d over a period of 6 d under day/night cycles.

### LUC assays

LUC activity of *pADH::LUC* reporter line was monitored in 8-day-old seedlings grown into a 96-well white plate (Thermo-Fisher Scientific) under growth conditions described above. Twenty-four hours before treatment, a solution of 5 mM of D-luciferin (Biosynth) was added to each well (20 μL per well), as previously described (Triozzi et al. 2024). The Tristar 5 multimode reader (Berthold Technologies, Germany) was used to measure the Firefly LUC activity by recording the total bioluminescence emitted in 5 s. *In vivo* LUC activity imaging of *p5xHRPE::LUC* reporter line was performed on 17-day-old plants using a NightSHADE equipment (Berthold Technologies, Germany). A solution of 5 mM of D-luciferin was sprayed on *p5xHRPE::LUC* leaves 24 h before starting the treatment. Indigo software was used to measure and quantify LUC activity every 1 h and with the following parameters: delayed time before acquisition was 120 s, 180 s of exposure time and binning was 4 × 4. The enzymatic activity of Nano Luciferase was quantified after recording green fluorescent protein (GFP) signal and following manufacturer's instructions (Promega). Total protein extraction from each seedling was performed using 150 μL of Passive Lysis Buffer (Promega). One microliter of protein extract was than incubated with 30 μL of Nano-Glo Luciferase Assay System before measuring luciferase activity. The Tristar 5 multimode reader (Berthold Technologies, Germany) was used to measure nLUC activity by recording the total bioluminescence emitted in 5 s.

### Gene expression analysis

Fifteen 8-day-old Arabidopsis seedlings were collected per biological replicate for RNA extraction. Total RNA was extracted using the Macherey-Nagel NucleoSpin RNA isolation kit according to the manufacturer's protocol. cDNA synthesis with Maxima cDNA Synthesis (Thermo-Fisher Scientific) and real-time quantitative PCR were performed as described previously (Loreti et al. 2020). *UBIQUITIN10* (At4g05320) served as the housekeeping gene for internal normalization, and relative expression levels were calculated using the 2^−ΔΔCT^ method. Primers used for qPCR analyses are listed in Table S4.

### Western blot analysis

Before total protein extraction, the chemical treatments described above were carried out. Subsequently, fifteen 8-day-old Arabidopsis seedlings were harvested in liquid nitrogen, and proteins were extracted from 100 mg of fresh tissue with 300 μL of protein extraction buffer (50 mM Tris-HCl pH 7, 1 mM EDTA pH 8, 100 mM NaCl, 2% SDS, and 0.05% Tween-20 with Protease Inhibitor Cocktail, Sigma). To analyze ERFVII stability after CQD treatment over 24 h, 3-week-old RAP2.3^3xHA^ plants were sprayed with either MOCK or CQD (100 μM). Leaves from three different plants were collected in liquid nitrogen at each time point and proteins were extracted from 100 mg of fresh tissue with 300 μL of protein extraction buffer. After spinning for 3 min at 14,000 RPM at 4 °C and recovering the supernatant, protein content was quantified using the Pierce BCA Protein Assay Kit (Thermo-Fisher Scientific). Western blot of RAP2.3^3xHA^ protein was performed as previously described (Triozzi et al. 2024).

Western blot of ADH and PDC proteins was performed by using polyclonal anti-ADH (Cat. n. AS10 685, Agrisera) and anti-PDC (Cat. n. AS10 691, Agrisera) antibodies together with a goat anti-Rabbit IgG horseradish peroxidase-conjugated secondary antibody (Cat. n. AS09 602, Agrisera) following manufacturer's instructions.

### GFP imaging

For GFP imaging, 8-day-old seedlings, grown in 96-well transparent plates (SPL Life Sciences), were treated by adding 30 µL at a concentration of 100 µM of the bioactive molecule to each well. Fluorescence emitted by GFP was then recorded at specific time points. GFP signal from *pbHLH38::3xGFP-nLUC* seedlings was detected using a Leica M205-FA thunder imaging system, using GFP filters (excitation: 470/40 nm, emission: 525/50) and an exposure time of 1.3 s. LAS X software was used to analyze and export images.

### *In vitro* AtPCO4 inhibition assays

To evaluate the effect of CQD on AtPCO4 activity, 0.4 µM recombinant AtPCO4 produced as described previously (Dirr et al. 2023) was incubated with 0.1 to 1,000 µM of CQD (dissolved in acetonitrile) for 5 min at 25 °C before exposure to 20 µM RAP2.12_2-15_ peptide, 5 mM TCEP and 1 mM ascorbate, in a reaction buffer of 50 mM Bris-Tris propane (pH 7.5) and 0.4 M NaCl. After 1 min, reactions were quenched with 5% formic acid. Samples were analyzed using RapidFire chromatography coupled with QTOF mass spectrometry, as described previously (Dirr et al. 2023). AtPCO4-catalysed oxidation of RAP2.12_2-15_ was quantified by comparing the areas underneath the product and substrate ions extracted from the total ion current chromatogram. Data were analyzed using GraphPad Prism. An equivalent volume of CQD was added to each sample such that they all contained 2.5% acetonitrile. 2.5% acetonitrile was also added to positive controls (without CQD) and results normalized to the positive control to account for any acetonitrile induced inhibition.

### *In silico* AtPCO4 docking experiments

AutoDockTools (version 1.5.7) provided as part of MGLTools (https://ccsb.scripps.edu/mgltools/downloads/) and AutoDock4 (version 4.2.6) (https://autodock.scripps.edu/download-autodock4/) developed in the Molecular Graphics Lab at The Scripps Research Institute were installed for use to simulate CQD docking. The 3D structure of CQD was downloaded from the PubChem database (https://pubchem.ncbi.nlm.nih.gov/) and AtPCO4 (PDB code: 6s7e, resolution 1.82 Å) was retrieved from RCSB Protein Data Bank (https://www.rcsb.org/). Both were prepared using AutoDockTools. Docking experiments were performed using default settings including energy range = 4, exhaustiveness = 8, and a grid box centered on the Fe atom (center coordinates: *x* = 33.429, *y* = 34.921, *z* = 18.621). Results were visualized using PyMOL.

### Waterlogging and submergence experiments

To test plant tolerance to waterlogging and submergence, 20-day-old Arabidopsis plants growing in pots were treated with 100 µM chlorquinadol or DMSO (MOCK) by leaf spraying and soil drenching with 1 mL of solution 24 h before the beginning of the waterlogging or submergence experiment, which was performed as previously described (Castellana et al. 2024). Three-week-old Arabidopsis plants were waterlogged and maintained under these conditions for 12 d. Twenty-five-day-old Arabidopsis plants were completely submerged in darkness and maintained under these conditions for 4 d. As a control treatment under normoxic conditions, plants sprayed with either MOCK or CQD solution were kept in the dark for 4 d. After this time, plants were allowed to recover under a photoperiod of 16 h/8 h light/dark cycles and light intensity of 150 μmol m^−2^ s^−1^ PPFD to induce flowering and seed production. Leaf color index (HUE) and leaf area at the selected time points were evaluated as previously described (Ventura et al. 2020). Seeds from each plant were harvested to quantify the total plant yield.

## Results

### Chemical genetic screening identifies inducers of hypoxia responses in Arabidopsis

**Figure 2:**
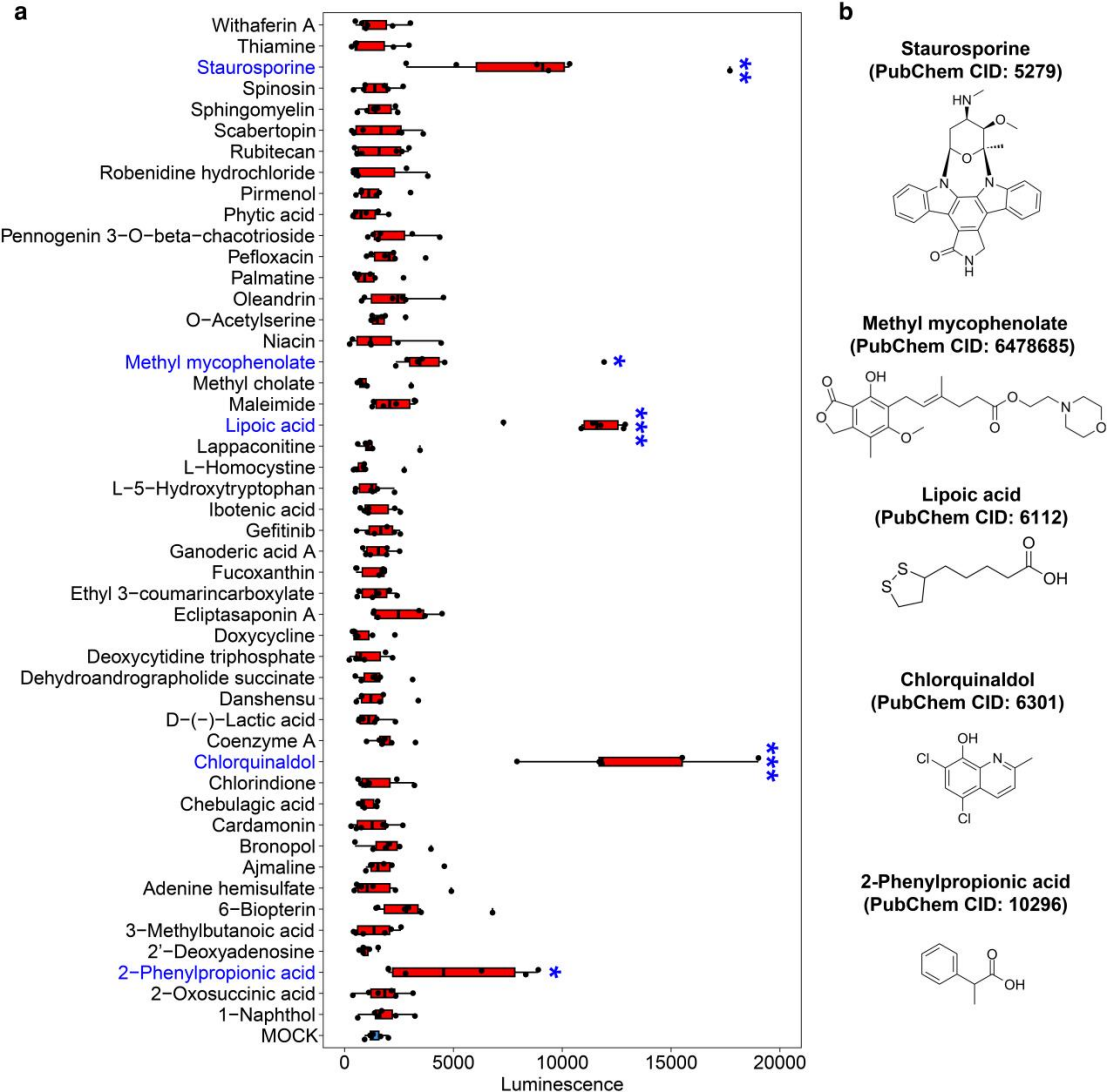
Validation of the high-throughput chemical genetic screen output identifies 5 chemical inducers of *pADH::LUC*. a) Luminescence signal from *pADH::LUC* Arabidopsis seeldings after 4 h treated with the 48 molecules identified by the high-throughput chemical genetic screen as putative chemical inducers of hypoxia responses. Student's *t*-test was performed (*n* = 6 biological replicates), and asterisks indicate statistically significant differences between MOCK and bioactive molecules inducing *pADH::LUC* activity (*P < 0.05, **P < 0.01, ***P < 0.001). b) Chemical structures of STS, MM, LA, CQD, and 2-phenylpropionic acid are shown.

To identify bioactive molecules with the potential to activate hypoxia responses in plants, we performed a high-throughput forward chemical genetic screening in *A. thaliana* harboring a firefly LUC-based reporter constituted by *ADH* promoter (*pADH::LUC*), which has been previously characterized as a reliable tool for monitoring the induction of anaerobic responses in plants (Brunello et al. 2024). *pADH::LUC* Arabidopsis seedlings grown in 96-well white plates (Fig. 1) were used to screen a large chemical library containing a total of 2,237 bioactive molecules isolated from animals, plants and microorganisms (Table S1). An initial screening was conducted on 8-day-old *pADH::LUC* seedlings, in which each seedling was treated with a molecule, and luminescence quantified after 4 h (Fig. 1). To consider a molecule as a potential chemical inducer of *pADH::LUC* activity, we established a threshold value based on the luminescence emitted by *pADH::LUC* Arabidopsis seedlings treated with bortezomib, an inhibitor of proteasome, that leads to ERFVII stabilization and activation of their hypoxia-inducible target genes (Zubrycka et al. 2023). Using this threshold, 60 out of 2,237 molecules (∼2.7% of the chemical library) resulted as potential inducers of *pADH::LUC* activity (Table S2). Among these chemicals, we identified 12 molecules previously shown to have a detrimental effect on plant growth or to be allelochemicals (Table S2), which were excluded from further validation. This analysis narrowed our list to 48 chemicals that were re-tested to confirm or not their ability to induce *pADH::LUC* activity. Only five molecules were confirmed to induce *pADH::LUC* reporter: staurosporine (STS), methyl mycophenolate (MM), lipoic acid (LA), CQD, and 2-phenylpropionic acid (Fig. 2a and b). STS was isolated from a soil-dwelling microorganism (*Lentzea albida*) and is an alkaloid with their typical bis-indole chemical structure (Fig. 2b). STS is a potent protein kinase C inhibitor that can induce apoptosis and possess insecticidal properties (Zhang et al. 2016; Ōmura et al. 2018). According to its function, Arabidopsis seedlings treated with STS died after 72 h (Figure S1). The activation of *pADH::LUC* reporter by STS might be associated to the generation of reactive oxygen species (ROS) generated during STS-induced apoptosis (Gil et al. 2003). ROS burst after STS application would inhibit PCO activity as shown recently (Akter et al. 2026) and, thus, activating *ADH* transcription through ERFVII stabilization. Our screen also found MM as chemical inducer of *pADH::LUC* reporter (Fig. 2a and b). MM is a derivative of mycophenolate, an inhibitor of inosine monophosphate dehydrogenase that suppresses T- and B-lymphocyte proliferation, which makes this molecule an effective immunosuppressant in transplantation (Allison and Eugui 2000). 2-PPA has a similar chemical structure to phenylacetic acid (Fig. 2b), which is a member of the auxin family (Hladík et al. 2025), phytohormones crucial for many aspects of plant growth, development, and in response to abiotic stresses (Sharma et al. 2015; Vanneste et al. 2025). LA is a known modulator of mitochondrial metabolism as it is an essential cofactor for the E_2_ catalytic component of a-ketoglutarate dehydrogenase complexes located in the mitochondria (Packer and Cadenas 2011; Solmonson and DeBerardinis 2018). CQD, characterized as 5,7-dichloro-2-methyl-8-hydroxyquinoline, is a chlorinated derivative of the chelating agent 8-hydroxyquinoline (Fig. 2b), an antibacterial agent known for its ability to chelate metal ions (Prachayasittikul et al. 2013).

Since STS and MM showed high plant toxicity and lowest activation of *pADH::LUC* among the molecules identified, respectively, only 2-PPA, LA, and CQD were selected for further investigation as chemical inducers of hypoxia responses.

### CQD induces hypoxia responses through ERFVII-dependent and independent pathways

**Figure 3:**
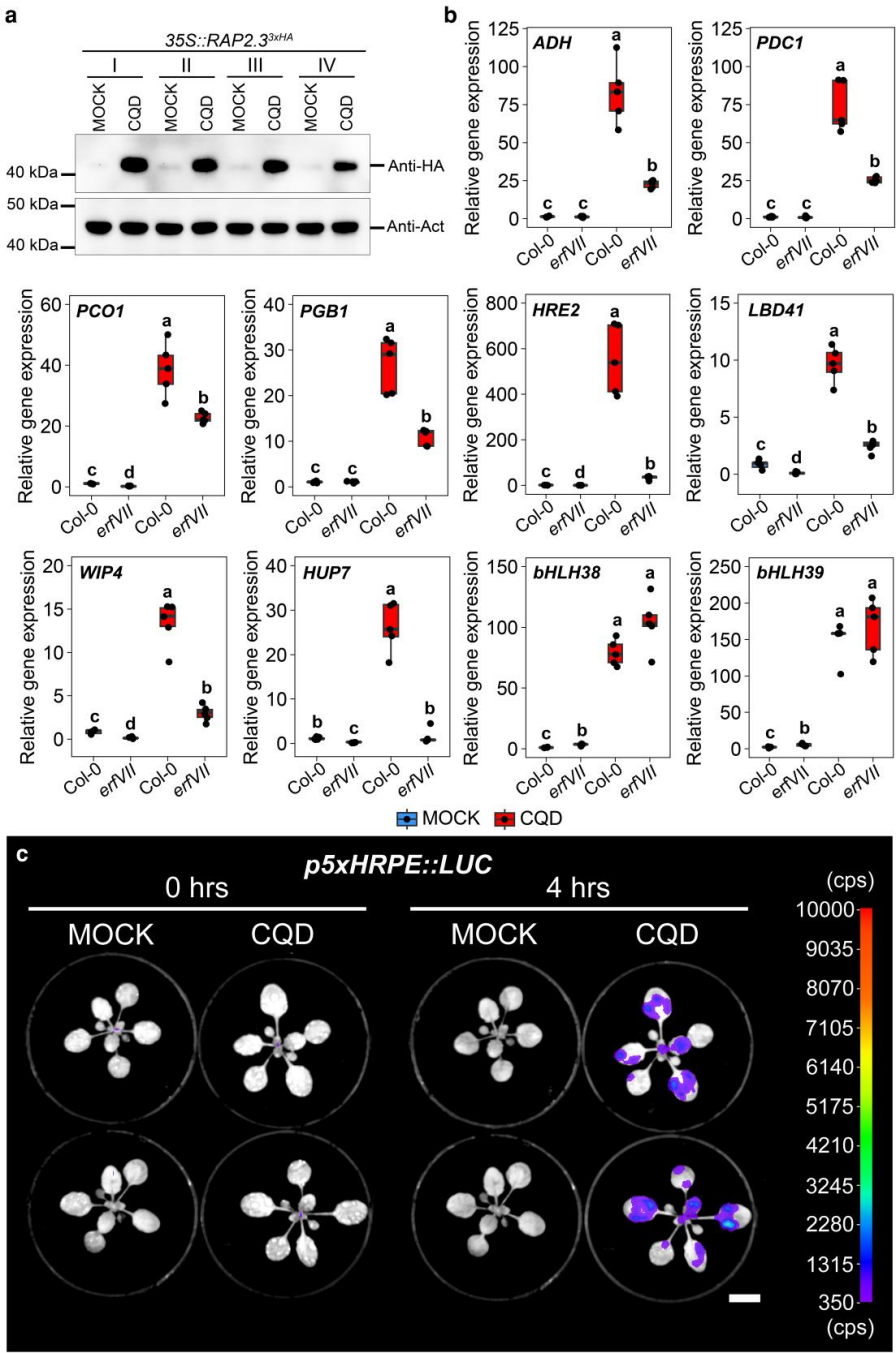
CQD induces hypoxia-responsive gene expression through ERFVIIs. a) Western blot analysis of RAP2.3^3xHA^ after 4 h of CQD or mock treatment performed on the *35S::RAP2.3^3xHA^* Arabidopsis transgenic line (four independent biological replicates are shown). b) Gene expression analysis of hypoxia-responsive genes (*ADH*, *PDC1*, *PCO1*, *PGB1*, *HRE2*, *LBD41*, *WIP4*, and *HUP7*) and iron starvation marker genes (*bHLH38* and *bHLH39*) after 4 h of CQD treatment compared to MOCK. The mRNA levels were measured by RT-qPCR, and the data on the y-axis are expressed relative to MOCK. To calculate statistical significance, the Kruskal–Wallis test followed by post hoc Wilcoxon test with multiple testing correction (Benjamini–Hochberg) was carried out, and groups with significant differences (*P*-value < 0.05) are indicated with different letters (*n* = 5 biological replicates). Boxplots center line represents the median. The box extends from the 25th to 75th percentiles, while the whiskers indicate the 1.5 times the interquartile range. Points outside the whiskers are outliers. c) Representative pictures of 17-day-old *p5xHRPE::LUC* plants sprayed with MOCK or CQD solution after 0 (ZT4) and 4 h (ZT8). Luminescence signal emitted by *p5xHRPE::LUC* plants is shown as counts per second. White scale bar = 1 cm.

To investigate the mode of actions of 2-PPA, LA, and CQD as chemical inducers of *pADH::LUC* reporter, we tested their ability to stabilize RELATED TO APETALA 2.3 (RAP2.3), a member of ERFVIIs, by using the previously characterized *35S::RAP2.3^3xHA^* Arabidopsis transgenic line (Gibbs et al. 2014). Western blot analysis of RAP2.3 abundance after 2-PPA, LA, or CQD treatments revealed that only CQD strongly stabilized RAP2.3 after 4 h under normoxic conditions (Figure S2 and Fig. 3a). Gene expression analysis of several hypoxia-responsive genes (HRGs; Mustroph et al. 2009) in the wild-type and pentuple mutant *erfVII* (in which all ERFVIIs are mutated; Abbas et al. 2015) after CQD treatment, revealed that CQD treatments activates the expression of several HRGs and that ERFVII function is required to properly establish the induction of HRGs by CQD (Fig. 3b). Compared to CQD treatment, 2-PPA and LA treatments led to the activation of only *ADH*, *PDC1* and *HYPOXIA RESPONSIVE ERF2* (*HRE2*) among the analyzed HRGs (Figures S3 and S4), suggesting that these molecules preferentially activated genes involved in alcoholic fermentation. Interestingly, 2-PPA strongly induced *GRETCHEN HAGEN 3.3* (*GH3.3*) expression (Figure S3), an auxin-inducible gene, suggesting it may function also as an auxin-like molecule.

These results indicated that CQD, unlike 2-PPA and LA, is a general inducer of hypoxia responses and that the HRG induction by CQD required ERFVII function (Fig. 3a). However, several HRGs such as *ADH*, *PDC1*, *PCO1*, and *PHYTOGLOBIN 1* (*PGB1*) still showed a certain degree of activation in the *erfVII* mutant (Fig. 3b), suggesting that ERFVII-independent pathways might also contribute to the activation of HRGs after CQD treatment. Activation of hypoxia responses also resulted in increased abundance of proteins involved in anaerobic metabolism, such as ADH and PDC, after 4 h of CQD treatment (Figure S5a–d). Notably, the increase in PDC abundance was even higher than that observed after 4 h of hypoxia (Figure S5d).

Since CQD belongs to a class of metal chelators, we also analyzed whether CQD treatment could affect cellular iron homeostasis by analyzing the expression of iron starvation marker genes, such as *bHLH38* and *bHLH39* (Wang et al. 2007). CQD treatment strongly induced *BASIC HELIX-LOOP-HELIX 38* (*bHLH38*) and *bHLH39* expression compared with the control condition (MOCK) in both wild-type and *erfVII* mutant (Fig. 3b), indicating that CQD application mimics an iron-depletion condition, which can stabilize ERFVIIs in Arabidopsis (Zubrycka et al. 2023; Telara et al. 2026). Moreover, as a readout of higher ERFVII activity due to their stabilization after CQD treatment, we analyzed the activation of a synthetic hypoxia transcriptional reporter constituted by five tandem repetitions of the hypoxia-responsive promoter element, which is bound by the ERFVII RAPs-type (Gasch et al. 2016), and LUC as reporter gene (*5xHRPEp:LUC*) (Brunello et al. 2024). After spraying a MOCK or CQD solution on *p5xHRPE::LUC* plants, luminescence was recorded every hour for 24 h using Nightshade equipment (Movie S1). According to activation of HRGs by CQD, *p5xHRPE::LUC* reporter was induced after 4 h in CQD-sprayed plants compared to MOCK-treated plants (Fig. 3c), with a peak of activity after 6 h (Figure S6). Interestingly, CQD-induced *p5xHRPE::LUC* activation was reversible and completely turned off after 15 h of treatment (Figure S6). This temporal pattern was mirrored by RAP2.3^3xHA^ protein levels, showing a gradual decline over 24 h following CQD-induced stabilization (Figure S7). Therefore, CQD induces hypoxia responses by stabilizing ERFVIIs in a reversible manner.

### CQD inhibits PCO

**Figure 4:**
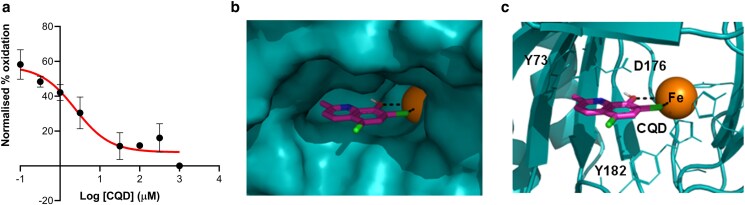
Inhibition of recombinant AtPCO4 by CQD. a) AtPCO4 activity toward a RAP2_2-15_ peptide substrate is inhibited by CQD in a dose-dependent manner; 0.4 µM AtPCO4 was preincubated with CQD for 5 min prior to exposure to 20 µM RAP2_2-15_ for 1 min at 25 °C. RAP2_2-15_ oxidation was normalized against a positive control containing acetonitrile solvent only. Data are presented as mean ± SD (*n* = 3). b, c) Representations of an AutoDock4 modeled structure of AtPCO4 (PDB 6S7E, teal) in complex with CQD (magenta, sticks). Active site Fe is represented as a sphere. A surface representation of AtPCO4 (b) suggests CQD binds in the substrate binding site; a cartoon representation of AtPCO4 (c) suggests CQD chelating the active site Fe (distances: Fe-CQD 8-OH = 3.8 Å, Fe-CQD 7-Cl = 3.4 Å).

The CQD's ability to chelate metal ions, which are important enzyme cofactors such as iron for PCOs (Weits et al. 2014; White et al. 2017, 2018) suggested that CQD could inhibit PCOs through the chelation of the cation Fe^2+^ and explain ERFVII stabilization and activation of hypoxia responses under normoxic conditions. To test whether CQD could inhibit PCO activity, we assessed the effect of CQD on the activity of recombinant AtPCO4, which is the most active enzyme of the five AtPCOs (White et al. 2018) and previously used to identify bioactive molecules as PCO inhibitors (Lavilla-Puerta et al. 2023). 0.4 μM AtPCO4 was preincubated with 0.1 to 1,000 µM of CQD for 5 min before being exposed to the Cys-initiating N-terminus of RAP2.12_2-15_ for 1 min under standard assay conditions (Dirr et al. 2023). The activity of AtPCO4, measured as the percentage of RAP2.12_2-15_ oxidized over a range of CQD concentration, was significantly reduced in the presence of CQD in a dose-dependent manner, with a half-maximal inhibitory concentration (IC50) value under these conditions of 2.42 μM compared to the equivalent assay in the presence of solvent-only control (Fig. 4a). This result indicated that the hypoxia responses induced by CQD are caused by direct inhibition of PCO activity. While we do not have a crystal structure of an AtPCO4-CQD complex, we used AutoDock4 to predict the mode of CQD binding to a reported crystal structure of AtPCO4 (White et al. 2020) (PDB ID 6S7E). These results suggest CQD has the potential to bind in the AtPCO4 active site (Fig. 4b) including via direct interactions with the active site Fe via the 8-OH (3.8 Å) and 7-Cl groups (3.4 Å) (Fig. 4c), consistent with an Fe-chelation mode of inhibition.

### 8-Hydroxy-5-nitroquinoline and CQD are the 8-hydroxyquinoline derivatives inducing ERFVII-dependent hypoxia responses

**Figure 5:**
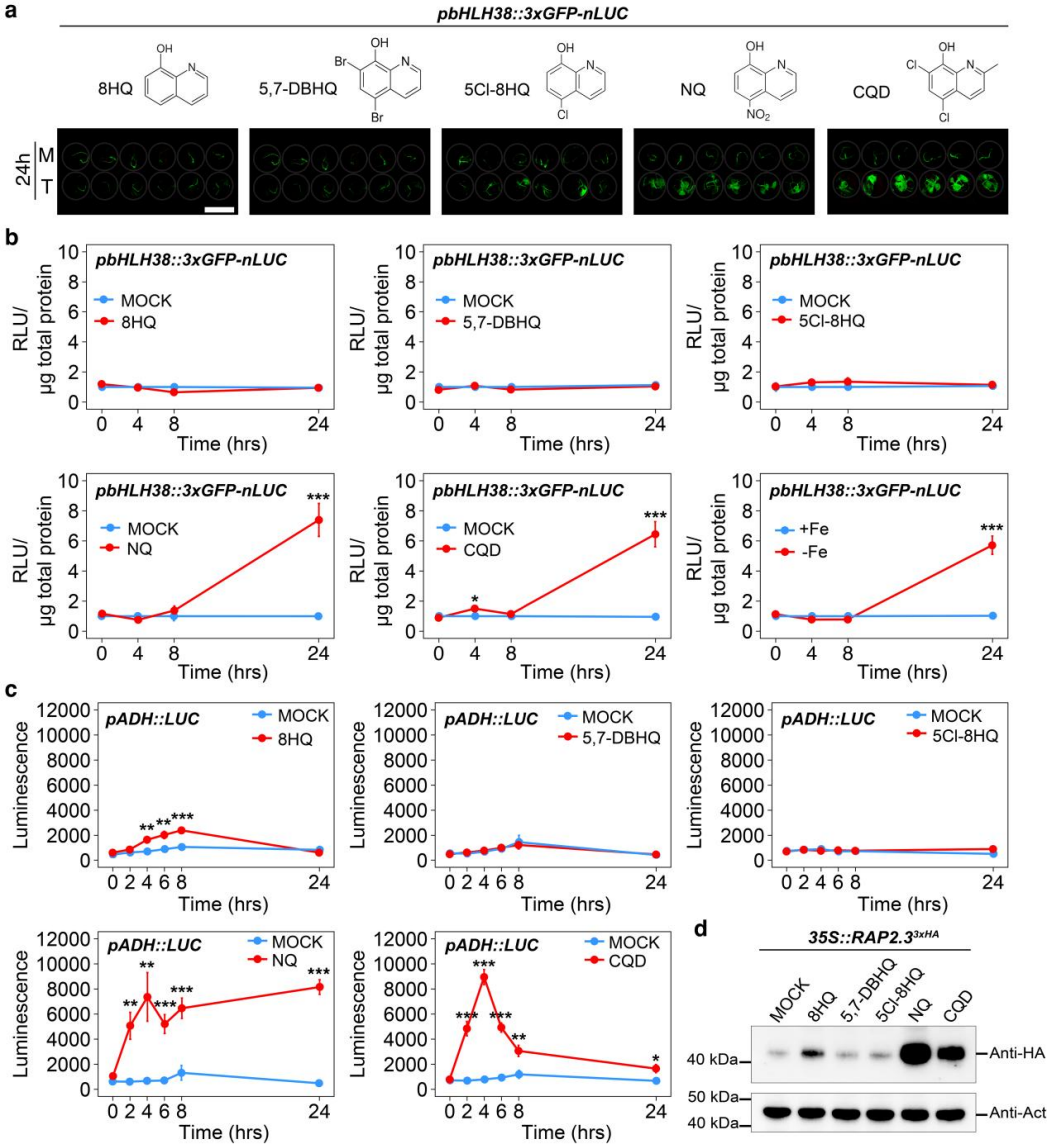
8HQ, NQ, and CQD induce hypoxia responses through ERFVII stabilization. a) GFP signal from 8-day-old *pbHLH38::3xGFP-nLUC* Arabidopsis seedlings after 24 h with a MOCK solution (M) or correspondent molecule treatment (T). White scale bar = 1 mm. b) Quantification of nLUC activity after total protein extraction from 8-day-old *pbHLH38::3xGFP-nLUC* Arabidopsis seedlings at different timepoints. Relative luminescence units (minimum value was = 1) were normalized on total protein content for each sample. Data are presented as mean ± SE (*n* = 6). *t*-test was performed and asterisks indicate ***P < 0.001. c) Quantification of luminescence (total luminescence units detected in 5 s) from 8-day-old *pADH::LUC* Arabidopsis seedlings at different timepoints. Data are expressed as mean ± SE (*n* = 6). Data are presented as mean ± SE (*n* = 6), *t*-test, *P-value < 0.05, **P-value < 0.01, ***P-value < 0.001. d) Western blot analysis of RAP2.3^3xHA^ abundance in 8-day-old *35S::RAP2.3^3xHA^* Arabidopsis seedlings after 4 h of control (MOCK) or 100 μM of 8HQ, 5,7-DBHQ, 5Cl-8HQ, NQ, and CQD treatments. Protein abundance of Actin 2 as loading control is shown.

We wondered whether the capacity to inhibit PCO activity and induce hypoxia responses was exclusive of CQD or was a shared feature among the derivatives of the 8-hydroxyquinoline. To test whether other 8-hydroxyquinoline derivatives could induce hypoxia responses through inhibition of PCO activity, we selected 8-quinolinol (8HQ), 5,7-dibromo-8-hydroxyquinoline (5,7-DBHQ), 5-chloro-8-quinolinol (5Cl-8HQ), and 8-hydroxy-5-nitroquinoline (NQ) together with CQD, which showed different electron-withdrawing substituents and therefore distinct structural and electronic variations (Fig. 5a). Since PCO inhibition by CQD is likely through its iron chelating activity, we first tested the iron chelating power of these 8-hydroxyquinoline derivatives. We did this by analyzing their effect on the activity of an iron starvation reporter line composed of the promoter of *bHLH38* controlling the expression of three enhanced green fluorescent protein in tandem (*GFP*), derived from *Aequorea victori*, fused to the nano LUC isolated from *Oplophorus gracilirostris* (*pbHLH38::3xGFP-nLUC*). Applications of 8HQ, 5,7-DBHQ, or 5Cl-8HQ were unable to induce *pbHLH38::3xGFP-nLUC*, as measured both by imaging GFP signal and quantifying nLUC activity (Fig. 5a and b). In contrast, NQ and CQD treatments led to a strong activation of *pbHLH38::3xGFP-nLUC* after 24 h, like that observed upon iron removal from the medium (Fig. 5a and b). 8HQ, 5,7-DBHQ, and 5Cl-8HQ showed low or no induction of the reporter *pADH::LUC* (Fig. 5c). According to their strong iron chelating activity, NQ and CQD strongly induce *pADH::LUC* activity (Fig. 5c), suggesting that NQ could also be an inhibitor of PCO activity. Interestingly, *pADH::LUC* induction persisted even after 24 h of NQ treatment, whereas CQD showed fast reversibility. The effect of these 8-hydroxyquinoline derivatives on *pADH::LUC* activity was reflected into ERFVII stability, where NQ and CQD treatments led to strong stabilization of RAP2.3 after 4 h (Fig. 5b). These results suggested that NQ and CQD have the strongest capacity to chelate iron among the 8-hydroxyquinoline derivatives, based on *pbHLH38::3xGFP-nLUC* and *pADH::LUC* activation. Other metal chelators, such as ferrozine and EDTA-Na_2_, were unable to induce *pADH::LUC* reporter (Figure S8) as observed for NQ and CQD treatments, indicating that the metal chelating capacity alone is not sufficient to be an effective PCO inhibitor and chemical inducer of hypoxia responses.

Together, these results show that among the 8-hydroxyquinoline derivatives tested, only NQ and CQD were the most effective chemical inducers of ERFVII-dependent hypoxia responses, although with different temporal dynamics.

### CQD treatment enhances plant tolerance to waterlogging and submergence

Given the transient activation of hypoxia responses by CQD treatment, we hypothesized that CQD pretreatment might represent an optimal strategy to induce a transient priming state that could help the plant to withstand subsequent flooding events. We first assessed whether CQD treatment negatively affects plant growth. To this end, shoots of 3-week-old Arabidopsis plants were sprayed with either MOCK or CQD solution and HUE and total leaf area, two parameters commonly used for phenotyping plants under normoxic and hypoxic conditions (Ventura et al. 2020), were recorded every 2 d over a period of 10 d under day/night cycles (Figure S9). Our results showed that neither HUE nor leaf area, as well as seed production, were affected by CQD compared with the control (Figure S9). On the other hand, analysis of root length and lateral root emergence after exposure of Arabidopsis seedlings to 4 h of CQD treatment revealed a shorter primary root and a lower number of lateral roots compared with the control (Figure S10). This result is in line with previous observations showing that Arabidopsis seedlings expressing a constitutively stabilized version of RAP2.12, a member of the ERFVII family, exhibited shorter primary root and reduced lateral root density (Shukla et al. 2019). Although these findings indicate that CQD may have a negative impact on root growth at the seedling stage, the same treatment did not affect the overall growth of adult plants (Figure S9), suggesting that CQD could be a suitable agent for chemically priming hypoxia responses in adult plants.

**Figure 6:**
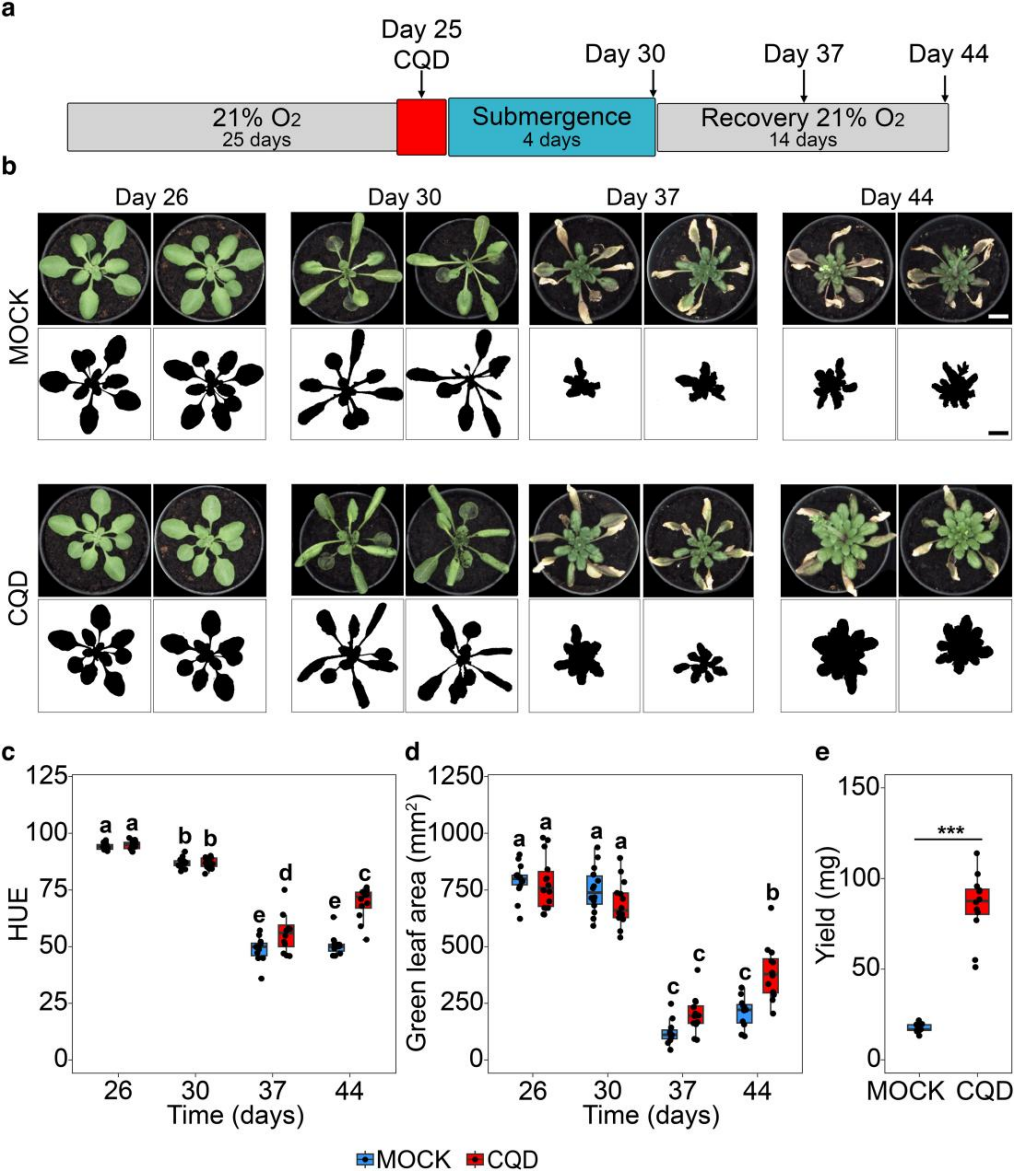
CQD treatment enhances plant tolerance to submergence. a) Schematic representation of the experimental design followed to test the priming effect induced by CQD pretreatment. Plants were grown under aerobic conditions for 25 d and then sprayed once on the leaf surface with either a CQD or MOCK solution at the end of the day (ZT10; red square). Plants were submerged 24 h after treatment. b) Representative pictures of plants before submergence (day 25), and during the recovery phase after 7 (day 37) and 14 d (day 44) under normoxic conditions. Scale bar = 1 cm. The selection of green leaf area (in black) before and after submergence was used to measure the total photosynthetic leaf area. c) HUE index of MOCK- and CQD-treated plants before submergence (day 26) and after submergence (day 30) and following 1 and 2 wk of recovery under normoxic conditions. Sample sizes were *n* = 14 before and after submergence, and *n* = 11 (MOCK) and *n* = 12 (CQD) during the recovery phase due to plant mortality following submergence. Kruskal–Wallis test followed by post hoc Wilcoxon test with multiple testing correction (Benjamini–Hochberg) was carried out to calculate groups with significant differences (*P*-value < 0.05), which are indicated with different letters. d) Quantification of total green leaf area before and after submergence. Green leaf area before and after submergence was extracted from each plant (shown in black) and quantified. Green leaf area after submergence was calculated from leaves emerged during the recovery phase. One-way ANOVA followed by Tukey HSD test was carried out to calculate groups with significant differences (*P*-value < 0.05), which are indicated with different letters. e) Seed production, measured as total harvested seeds per plant, from MOCK- and CQD-treated plants. *t*-test, ***P-value < 0.001. Boxplots center line represents the median. The box extends from the 25th to 75th percentiles, while the whiskers indicate the 1.5 times the interquartile range. Points outside the whiskers are outliers.

We therefore investigated whether CQD pretreatment enhances Arabidopsis tolerance to submergence. For this purpose, 25-day-old Arabidopsis plants were sprayed with a MOCK or a CQD solution and submerged in the dark or kept in the dark under normoxic conditions for 4 d (Fig. 6a and b; Figure S11). After 2 d of recovery under day/night cycles, CQD-treated plants kept in the dark showed earlier senescence on some old leaves as showed by lower HUE compared to MOCK-treated plants (Figure S11c). After submergence and 1 wk of recovery under normoxic conditions, HUE index and newly formed green leaf area were significantly higher in CQD-treated than MOCK-treated submerged plants (Fig. 6c and d). After 2 wk, CQD-treated submerged plants exhibited a stronger vegetative recovery than MOCK-treated plants, as showed by higher HUE index and green leaf area (Fig. 6c and d) and higher yield (Fig. 6e). Notably, CQD treatment did not improve the survival rate across different submergence durations compared to the MOCK-treated plants (Figure S12).

We next investigated whether CQD pretreatment could also improve plant tolerance to waterlogging. Using a similar experimental set-up as to the submergence experiment, 3-week-old Arabidopsis plants were sprayed with a MOCK or a CQD solution and waterlogged after 24 h (Figure S13a and b). After 12 d of waterlogging, HUE index and total leaf area were measured after 3 and 8 d of recovery (Figure S13a and b). CQD-treated plants exhibited a significantly higher HUE index than in MOCK-treated plants after 3 and 8 d of recovery in normoxic conditions (Figure S13c), although no difference in total leaf area was observed (Figure S13d). Notably, CQD-treated plants showed a significantly higher yield, as measured by total seed production, than MOCK-treated plants (Figure S13e), indicating a beneficial effect from CQD pretreatment on plant tolerance to waterlogging.

Together, CQD resulted in an effective chemical inducer of hypoxia responses and a modulator of PCO activity under normoxic conditions. These results demonstrated that CQD treatment prime plants before they experience hypoxia-associated stress, such as during waterlogging or submergence after flooding, ultimately improving their survival.

## Discussion

Genetic engineering of the PCO-branch of the N-Degron pathway in plants has proven to be an effective strategy to enhance plant tolerance and survival under low-oxygen stress during flooding (Dirr et al. 2025; Perri et al. 2025). Alternatively, modulation of the oxygen-sensing pathway could be achieved by using molecules that inhibit PCO activity and prime hypoxia responses (White and Flashman 2016; Lavilla-Puerta et al. 2023).

Using a plant chemical genetic approach, we performed a high-throughput screen of 2237 bioactive molecules using a hypoxia-responsive reporter, *pADH::LUC*, and identified 60 as potential chemical inducers of hypoxia responses in Arabidopsis. Among these molecules, 12 were previously described as herbicides or allelochemicals that negatively affect plant growth (Table S3). Moreover, STS, which is one of the five molecules confirmed to strongly induce *pADH::LUC* activity (Fig. 2), induced apoptosis in Arabidopsis (Figure S1). These results were in line with previous studies showing that the activation of fermentative metabolism occurs after herbicide treatments (Gil-Monreal et al. 2019). Thus, *pADH::LUC* reporter might also be exploited to identify novel herbicide-like molecules through high-throughput chemical genetic approaches.

In addition to molecules with herbicide-like function, we found 2-PPA as chemical inducer of *ADH* transcription (Fig. 2 and Figure S3). 2-PPA shares a similar chemical structure with the auxin phenylacetic acid (Hladík et al. 2025). Strong induction of the auxin-inducible gene *GH3.3* by 2-PPA (Figure S3) likely indicates that it functions as an auxin-like molecule. Previous studies have reported the interplay between hypoxia and auxin, especially in the context of root responses to hypoxia and lateral root development, where ERFVIIs antagonistically link oxygen-sensing to auxin signaling (Eysholdt-Derzsó and Sauter 2017; Shukla et al. 2019). The reason why 2-PPA treatment exclusively led to the activation of genes involved in alcoholic fermentation, such as *ADH* and *PDC1*, remains to be determined. Since auxin play central role in plant meristems (Vanneste et al. 2025), it can be speculated that 2-PPA treatment could interfere with auxin signaling, cell division and the establishment of hypoxic niches in the context of plant meristems (Shukla et al. 2019; Castellana et al. 2026), which eventually could lead to the activation of genes involved in anaerobic metabolism. Moreover, it remains unclear whether the capacity to induce genes involved in anaerobic metabolism is peculiar to 2-PPA or is shared by other members of the auxin family, and whether this activation occurs by direct binding of Auxin Response Factors to *ADH* and *PDC1* promoters (Tiwari et al. 2003).

Like 2-PPA, we identified LA as an inducer of *ADH* and *PDC1* (Figure S4). The fact that 2-PPA and LA mainly activate genes belonging to anaerobic metabolism among the hypoxia-responsive genes tested (Figures S3 and S4) indicates that the activation of alcoholic fermentation can be uncoupled from other hypoxia-dependent transcriptional responses. However, the mechanism by which these molecules generate this uncoupling remains unknown. LA can chelate several metal ions, including iron (Camiolo et al. 2019). However, LA treatment, as well as 2-PPA, did not induce a strong RAP2.3 stabilization (Figure S2), suggesting that its capacity to induce *ADH* transcription is not through PCO inhibition. LA is a cofactor of a-ketoglutarate dehydrogenases, such as pyruvate dehydrogenase complex (mtPDC) (Solmonson and DeBerardinis 2018). Exogenous application of LA inhibits pyruvate dehydrogenase kinase (PDK), which, in turn, inactivates mtPDC activity by phosphorylating three specific serine residues of E1 (Korotchkina et al. 2004). MtPDC is the gatekeeper that converts pyruvate into acetyl-CoA, which enters the mitochondrial tricarboxylic acid (TCA) cycle for ATP production via oxidative phosphorylation (Kaplon et al. 2013). Thus, by inhibiting PDK, LA treatment could lead to a more active mtPDC, which, in turn, promotes pyruvate utilization via cellular respiration. It has been reported that increased mtPDC stimulates cellular respiration and oxygen consumption, which can, in turn, generate transient hypoxia, explaining how LA could activate mainly genes involved in alcoholic fermentation (Marillia et al. 2003; Ohbayashi et al. 2019). While future studies will define the modes of action of 2-PPA and LA, their ability to primarily induce genes responsible for alcoholic fermentation makes these molecules interesting chemicals that can be exploited to uncouple anaerobic metabolism from the activation of other ERFVII-dependent hypoxia responses.

Beyond identifying new chemical inducers of hypoxia responses in Arabidopsis, our work investigates the mode of action of CQD, a molecule previously characterized as an antimicrobial agent (Bortolin et al. 2017; Bidossi et al. 2019). CQD treatment induced the expression of several HRGs and accumulation of enzymes responsible for alcoholic fermentation, such as ADH and PDC (Figure S5), due to its ability to stabilize ERFVIIs under normoxic conditions (Fig. 3). However, some HRGs still showed a partial activation in the *erfVII* mutant, suggesting that CQD might also promote the activation of ERFVII-independent pathways controlling HRG expression. Alternatively, the lack of a RAP2.2 knockout mutation in *erfVII* mutant (Giuntoli et al. 2017) could explain why residual activation of some HRGs is still observed after CQD treatment (Fig. 3). The ability of CQD to stabilize ERFVIIs depends on the inhibition of PCO enzymes, likely through the chelation of iron in their catalytic site as predicted by docking experiments (Fig. 4). CQD apparently fits in the AtPCO4 active site, potentially stabilized by active site residues (the CQD 7-Cl is 3.4 Å from both the active site Fe and the Tyr182 hydroxyl group). This may suggest that the interaction of CQD with the active site metal supports a competitive inhibition model, rather than inhibition by chelation removing the metal from the enzyme.

Although our biochemical and genetic analyses show that CQD inhibits PCO activity and stabilizes ERFVIIs, we cannot conclude that CQD acts as a highly specific PCO inhibitor. Indeed, CQD has recently been found to inhibit Methionine Synthase Reductase by directly interacting with its FAD-binding domain, thereby inhibiting fibroblast activation in mice (Yang et al. 2024). Moreover, 8-HQ compounds have been used as metal-binding pharmacophores in drug-discovery screens precisely because they inhibit many metalloenzymes, including 2-oxoglutarate and iron-dependent oxygenases (Hopkinson et al. 2013). Therefore, while CQD treatment could affect other biochemical processes, the inhibition of PCO coupled with the stabilization of ERFVIIs and activation of hypoxia responses resulted beneficial for plants that undergo hypoxic stress (Fig. 6). Interestingly, inhibition of PCO activity and induction of hypoxia responses cannot by simply achieved through chelation of its iron cofactor by using iron chelating agents (Figure S3), indicating that the capacity of a molecule to chelate metal ions is not sufficient to be an efficient inhibitor of PCO enzymes. Supporting this, our comparison analysis of 8-hydroxyquinoline derivatives revealed that only CQD and NQ were potent inducers of hypoxia responses through PCO inhibition (Fig. 5), suggesting that specific electron-withdrawing substituents on the 8-hydroxyquinoline structure may confer unique properties, making these chemicals strong PCO inhibitors.

Our results highlight the importance of high-throughput chemical genetic screens for identifying novel molecules that function as potent and effective inhibitors of PCO activity in plants. This is biologically relevant because hypoxia-mimicking molecules could induce a plant priming state and improve their ability to cope with subsequent flooding events, as demonstrated by this study. Indeed, a CQD pretreatment enhanced plant tolerance to waterlogging and submergence (Fig. 6 and Figure S11), reinforcing the idea that PCO activity inhibitors represent promising tools to modulate and enhance plant resilience to low-oxygen stress conditions. Importantly, identifying novel PCO inhibitors will also be crucial for identifying more efficient PCO enzymes across the plant kingdom (Chirinos et al. 2025), revealing the unique catalytic features of species-specific PCOs and thereby informing genetic engineering strategies to improve crop resilience to flooding.

## Supplementary Material

kiag505_Supplementary_Data

## Data Availability

The data underlying this article are available in the article and in its online supplementary material.
