# Supplementary material for: Chemical priming of hypoxia responses via PLANT CYSTEINE OXIDASE inhibition improves flooding tolerance
Source: Plant Physiol. 2026 Jul 14;201(3):kiag505. doi: 10.1093/plphys/kiag505 (PMC13418359; doi:10.1093/plphys/kiag505)
Supplement: kiag505_Supplementary_Data [file kiag505_supplementary_data.zip › Supplemental_Figures.pdf]

SUPPLEMENTARY FIGURES

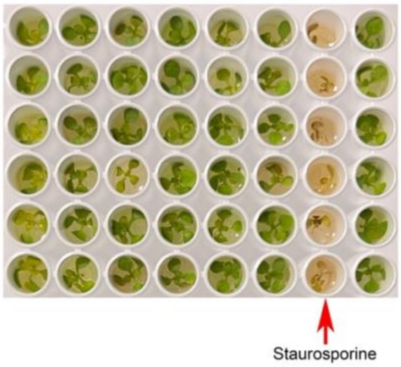

Figure S1. Staurosporine (STS) triggers apoptosis. Red arrow indicates Arabidopsis seedlings (entire column) after 72 hours of STS treatment.

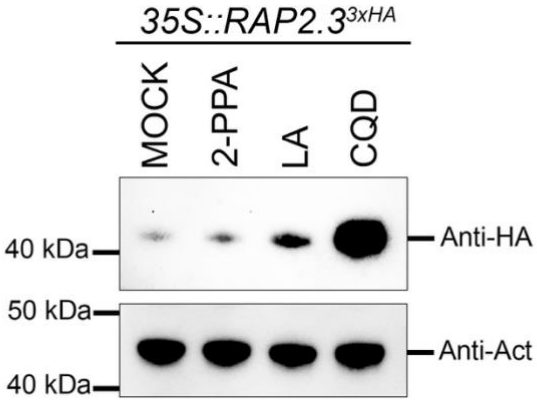

Figure S2. CQD treatment stabilizes RAP2.3. Western blot analysis of RAP2.3^{3xHA} abundance in 8-day-old *35S::RAP2.*3^{3xHA} Arabidopsis seedlings after 4 hours of control (MOCK) or 100 μM of 2-PPA, LA, and CQD treatments. Protein abundance of Actin 2 as loading control is shown.

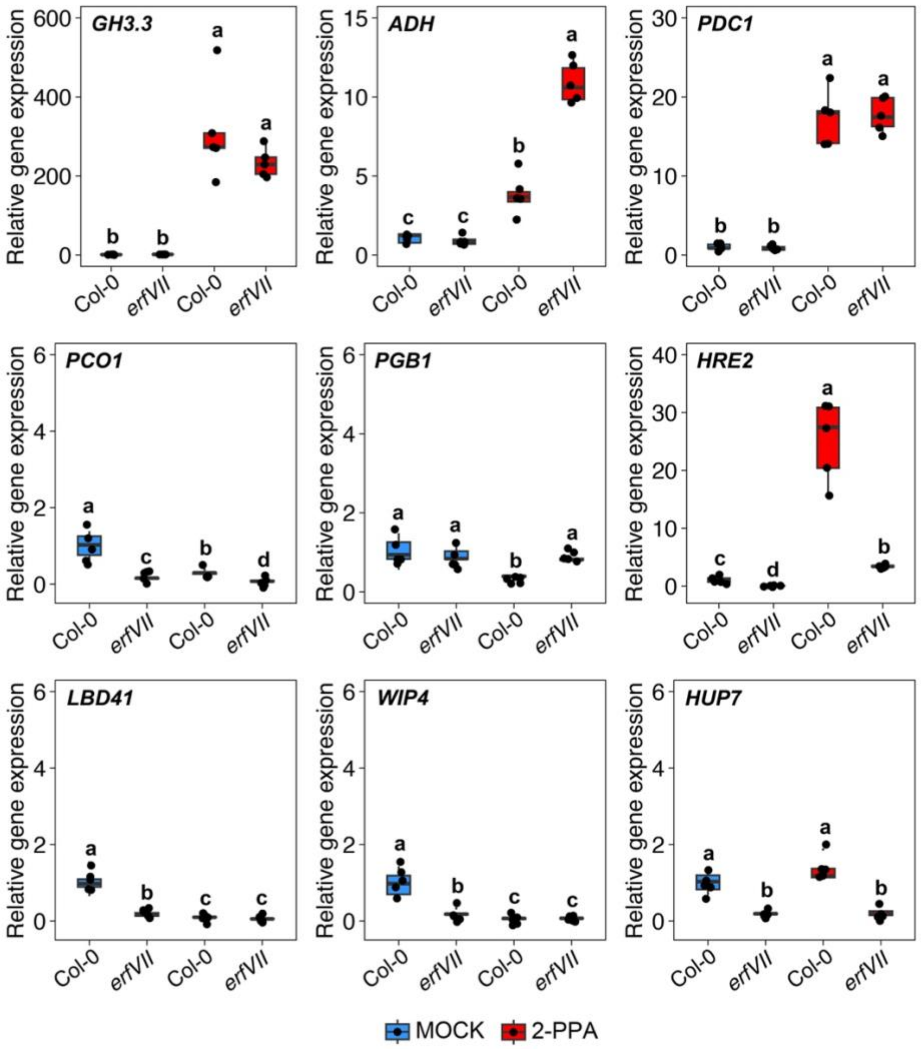

Figure S3. 2-phenylpropionic acid (2-PPA) treatment induces genes of alcholic fermentation. Gene expression analysis of an auxin inducible gene *GH3*.3 and hypoxia-responsive genes (*ADH*, *PDC1*, *PCO1*, *PGB1*, *HRE2*, *LBD41*, *WIP4* and *HUP7*) after 4 hours of 2-PPA treatment compared to MOCK. The mRNA levels were measured by RT-qPCR, and the data on the y-axis are expressed relative to MOCK. To calculate statistical significance, the Kruskal-Wallis test followed by post-hoc Wilcoxon test with multiple testing correction (Benjamini-Hochberg) was carried out, and groups with significant differences (p-value < 0.05) are indicated with different letters ($n = 5$ biological replicates). Boxplots center line represents the median. The box extends from the 25th to 75th percentiles, while the whiskers indicate the 1.5 times the interquartile range. Points outside the whiskers are outliers.

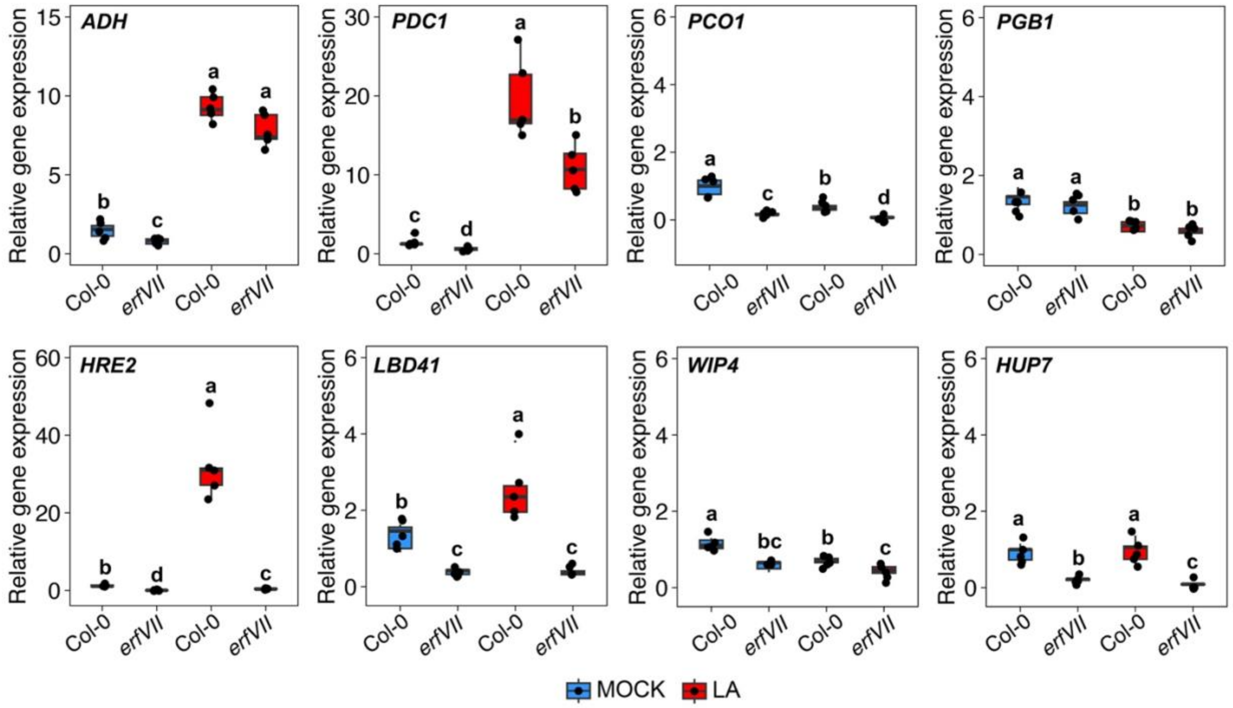

Figure S4. Lipoic acid (LA) treatment induces genes of alcholic fermentation. Gene expression analysis of hypoxia-responsive genes (*ADH*, *PDC1*, *PCO1*, *PGB1*, *HRE2*, *LBD41*, *WIP4* and *HUP7*) after 4 hours of LA treatment compared to MOCK. The mRNA levels were measured by RT-qPCR, and the data on the y-axis are expressed relative to MOCK. To calculate statistical significance, the Kruskal-Wallis test followed by post-hoc Wilcoxon test with multiple testing correction (Benjamini-Hochberg) was carried out, and groups with significant differences (p-value < 0.05) are indicated with different letters ($n = 5$ biological replicates). Boxplots center line represents the median. The box extends from the 25th to 75th percentiles, while the whiskers indicate the 1.5 times the interquartile range. Points outside the whiskers are outliers.

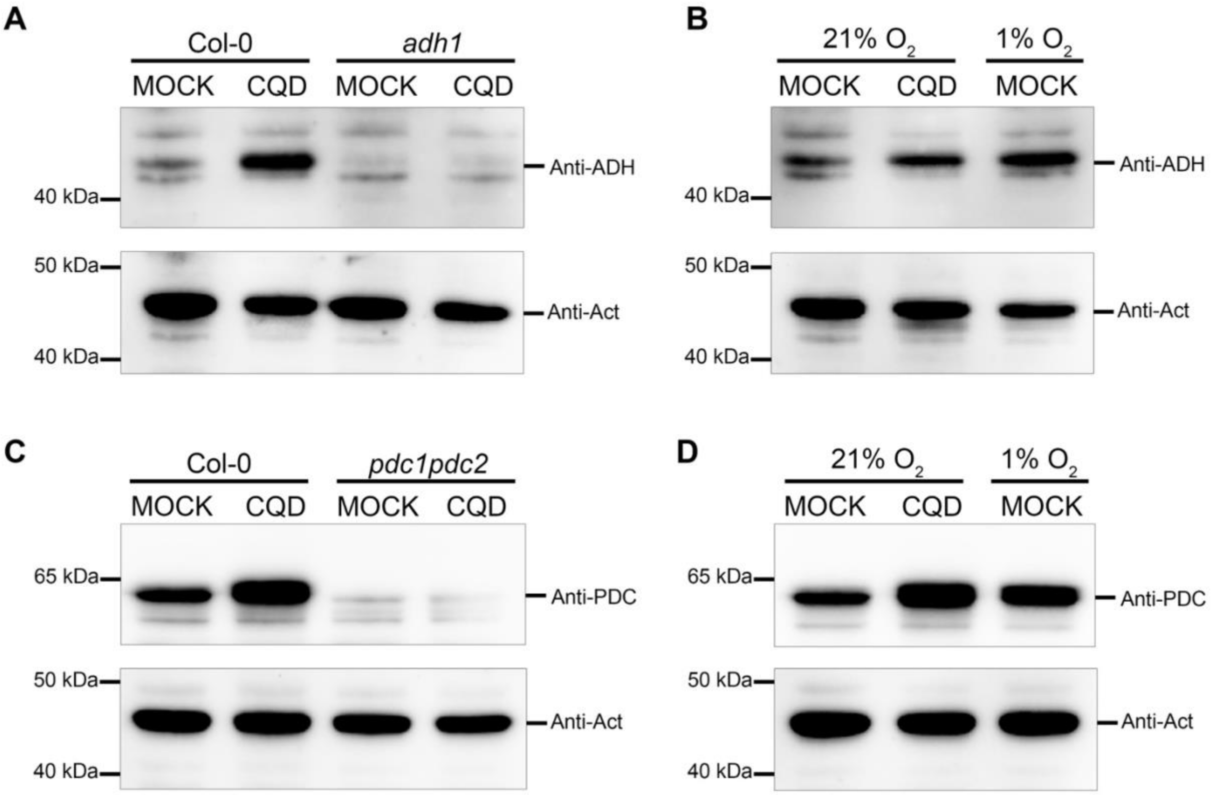

Figure S5. CQD treatment increases the abundance of ADH and PDC enzymes. (A) Western blot analysis of ADH1 protein abundance in 8-day-old Arabidopsis Col-0 and *adh1* mutant seedlings after 4 hours of MOCK treatment or 100 μM of CQD. (B) Western blot analysis of ADH1 protein abundance in 8-day-old Arabidopsis Col-0 seedlings after 4 hours of MOCK treatment at 21 and 1% of O_2 or 100 μM of CQD at 21% of O_2. (C) Western blot analysis of PDC protein abundance in 8-day-old Arabidopsis Col-0 and *pdc1pdc2* mutant seedlings after 4 hours of MOCK treatment or 100 μM of CQD. (D) Western blot analysis of PDC protein abundance in 8-day-old Arabidopsis Col-0 seedlings after 4 hours of MOCK treatment at 21 and 1% of O_2 or 100 μM of CQD at 21% of O_2.

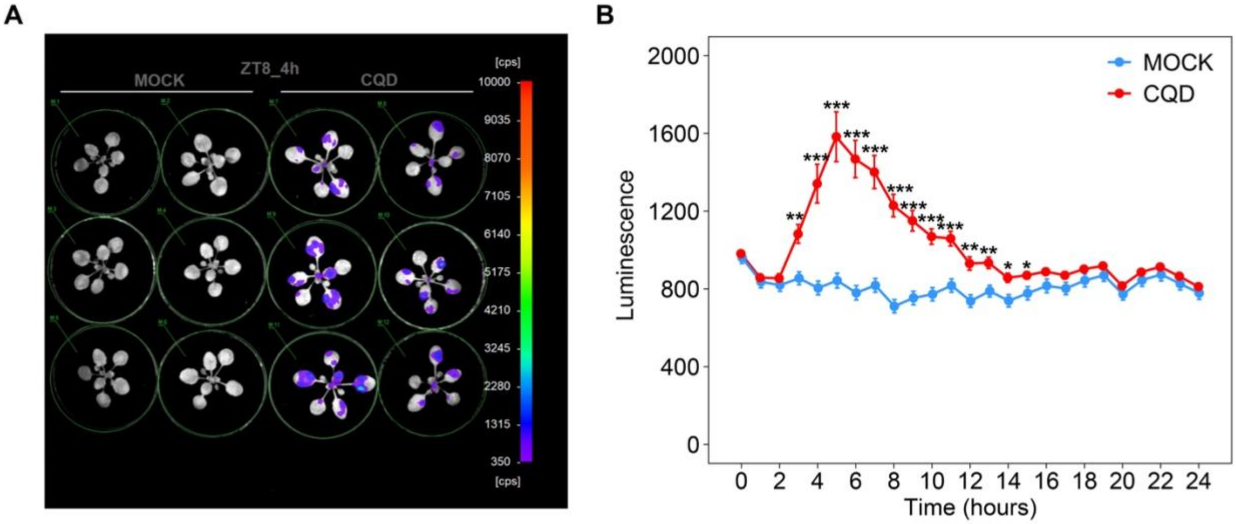

Figure S6. CQD treatment induces *p5xHRPE::LUC* activity under normoxic conditions. (A) *p5xHRPE::LUC* plants treated with MOCK or CQD after 4 hours at zeitgeber time 8 (ZT8), where ZT8 indicates 8 hours after lights turned on in a 12h/12h light-dark cycle. MOCK and CQD solutions were sprayed once onto the leaf surface. The area of the circle (green) was used for quantifying the luminescence emitted from each plant. (B) Quantification of luciferase activity expressed as counts per second (CPS) from *p5xHRPE::LUC* reporter line from time-lapse experiment (Movie S1). Quantification of CPS was carried out using the IndiGO. The data represent mean ± SE (n= 6). *t*-test was performed at each time point, and asterisks indicate statistically significant differences of *p5xHRPE::LUC* activity between MOCK and CQD treatments (*p-value <0.05, ** p-value <0.01, *** p-value <0.001).

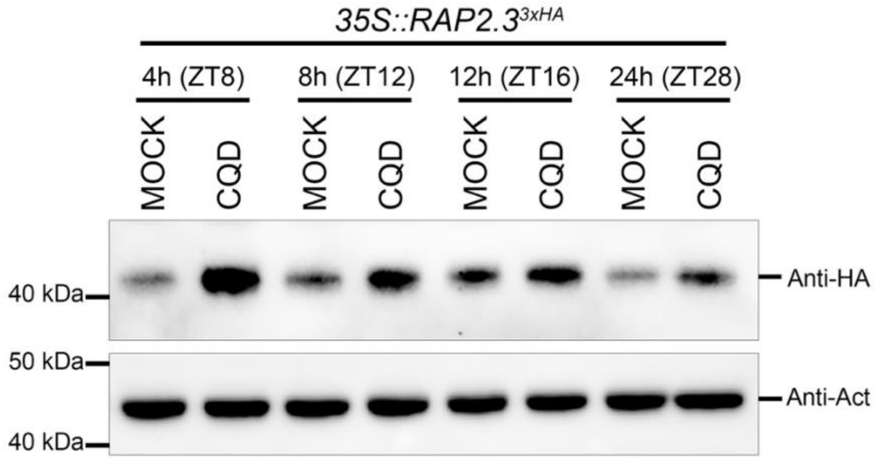

Figure S7. RAP2.3 is transiently stabilized by CQD treatment. Western blot analysis of RAP2.3^{3xHA} abundance in 3-week-old *35S::RAP2.3^{3xHA}* Arabidopsis plants after 4, 8, 12 and 24 hours of control (MOCK) or CQD treatment. Protein abundance of Actin 2 as loading control is shown. MOCK and CQD solutions were sprayed once onto the leaf surface at ZT4.

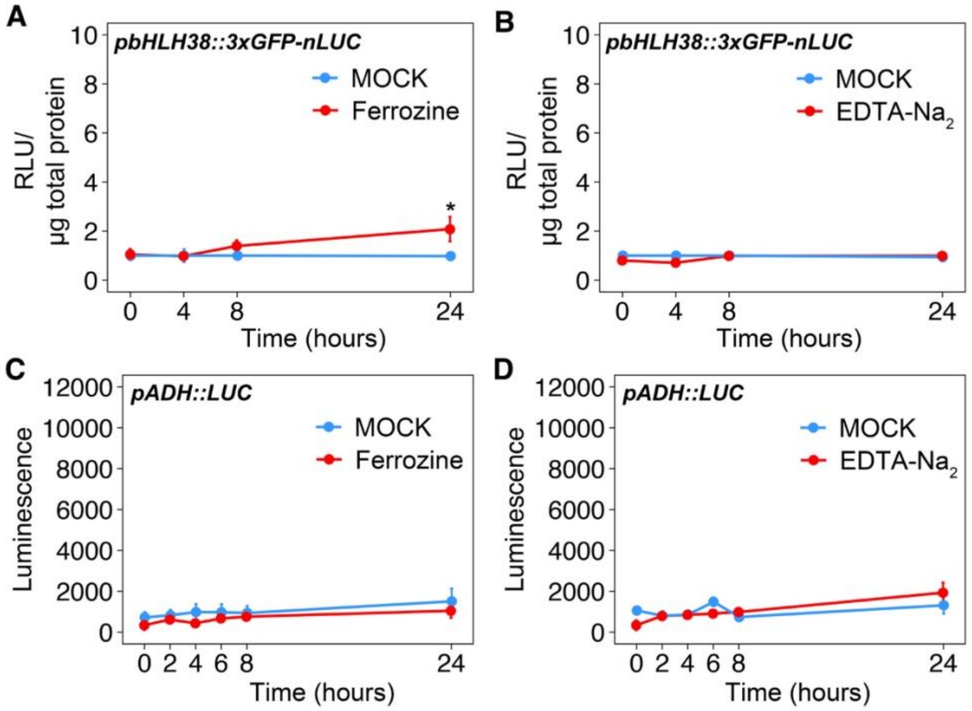

Figure S8. Iron or metal chelators were unable to induce hypoxia responses in plants. Quantification of nLUC activity after total protein extraction from 8-day-old *pbHLH38::3xGFP-nLUC* Arabidopsis seedlings at different timepoints after (A) ferrozine and (B) EDTA-Na_2 treatments. Relative luminescence units (RLU; minimum value was = 1) were normalized on total protein content for each sample. Data are presented as mean ± SE (n = 6). *t*-test, asterisk indicate *P-value < 0.05. Quantification of luminescence (luminescence units detected in 5 sec) from 8-day-old *pADH::LUC* Arabidopsis seedlings at different timepoints after (C) ferrozine (100 µM) and (D) EDTA-Na_2 treatments (100 µM). Data are expressed as mean ± SE (n = 6).

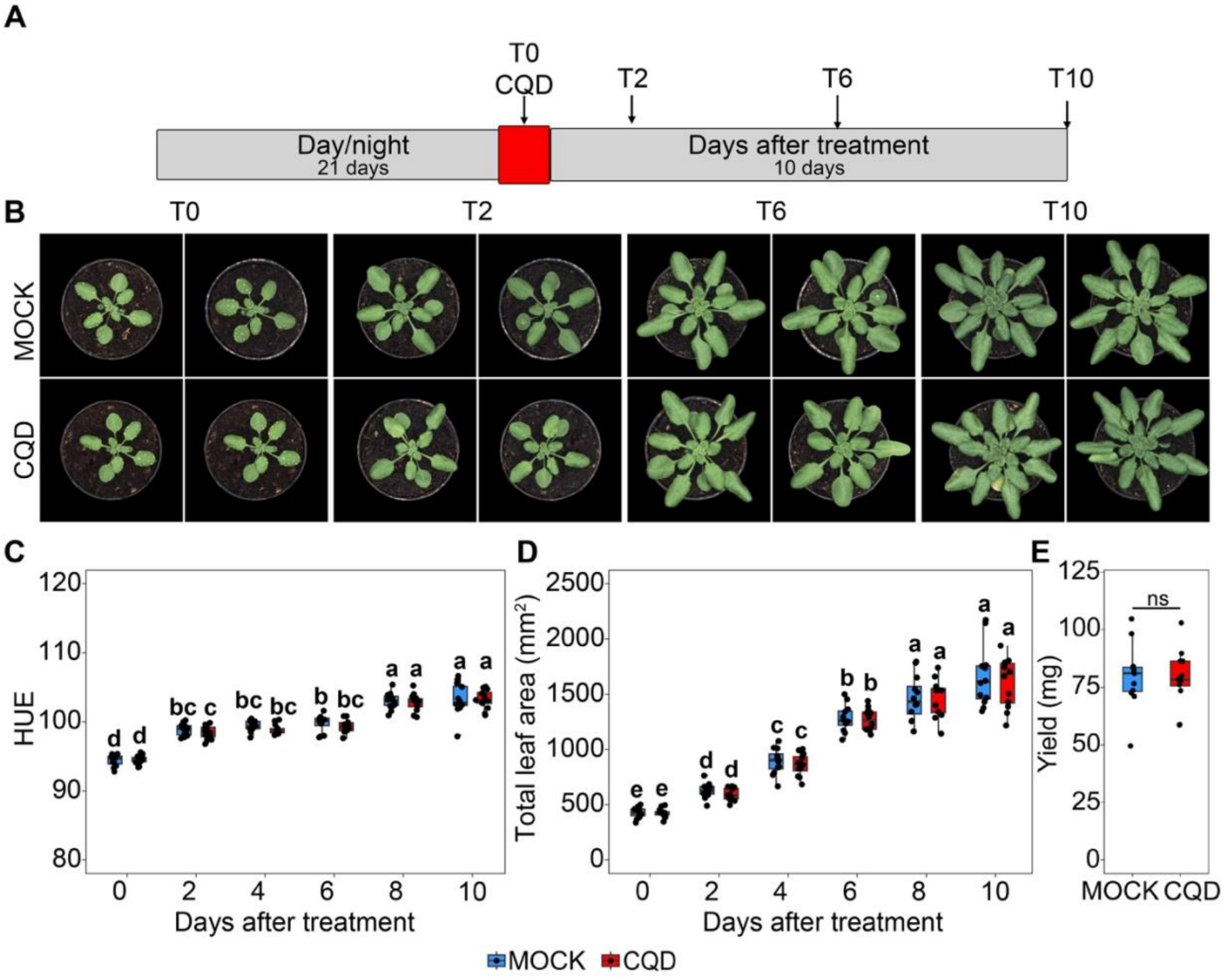

Figure S9. CQD treatment do not affect shoot growth and seed production. (A) Schematic representation of the experimental design followed to test whether CQD could negatively affect plant growth under standard growing conditions. MOCK and CQD solutions were sprayed once onto the leaf surface at the indicated time point (red square). (B) Representative pictures of 3-weeks-old Arabidopsis plants after MOCK and CQD treatment at different time points under day/night cycles. Scale bar = 1 cm. (C) HUE index of MOCK- and CQD-treated at different time points under day/night cycles and normoxic conditions (n=12). (D) Quantification of total leaf area after MOCK and CQD treatments at different time points under day/night cycles and normoxic conditions (n=12). Kruskal-Wallis test followed by post-hoc Wilcoxon test with multiple testing correction (Benjamini-Hochberg) was carried out in (C) and (D) to calculate groups with significant differences (p-value < 0.05), which are indicated with different letters. (E) Seed production, measured as total harvested seeds per plant, from MOCK- and CQD-treated plants. *t*-

test, p-value >0.05, not significant (ns). Boxplots center line represents the median. The box extends from the 25th to 75th percentiles, while the whiskers indicate the 1.5 times the interquartile range. Points outside the whiskers are outliers.

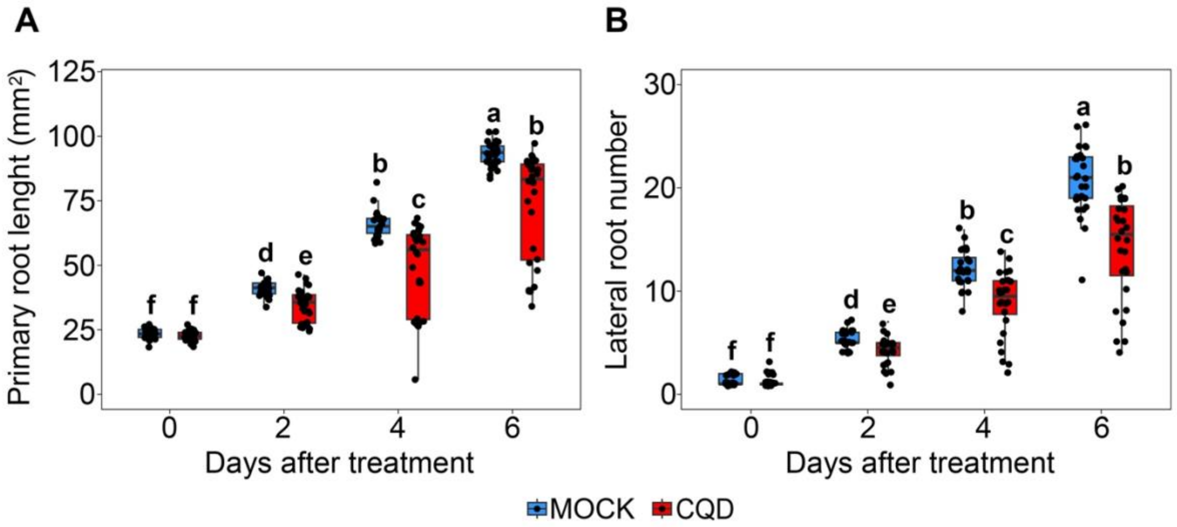

Figure S10. CQD treatment affects primary root length and lateral root number in Arabidopsis seedlings. (A) Primary root length and (B) number of lateral roots were measured after 4 hours of MOCK and CQD treatment (day 0) and after 2, 4 and 6 days (for each treatment n=28). Kruskal-Wallis test followed by post-hoc Wilcoxon test with multiple testing correction (Benjamini-Hochberg) was carried out in (A) and (B) to calculate groups with significant differences (p-value < 0.05), which are indicated with different letters. Boxplots center line represents the median. The box extends from the 25th to 75th percentiles, while the whiskers indicate the 1.5 times the interquartile range. Points outside the whiskers are outliers.

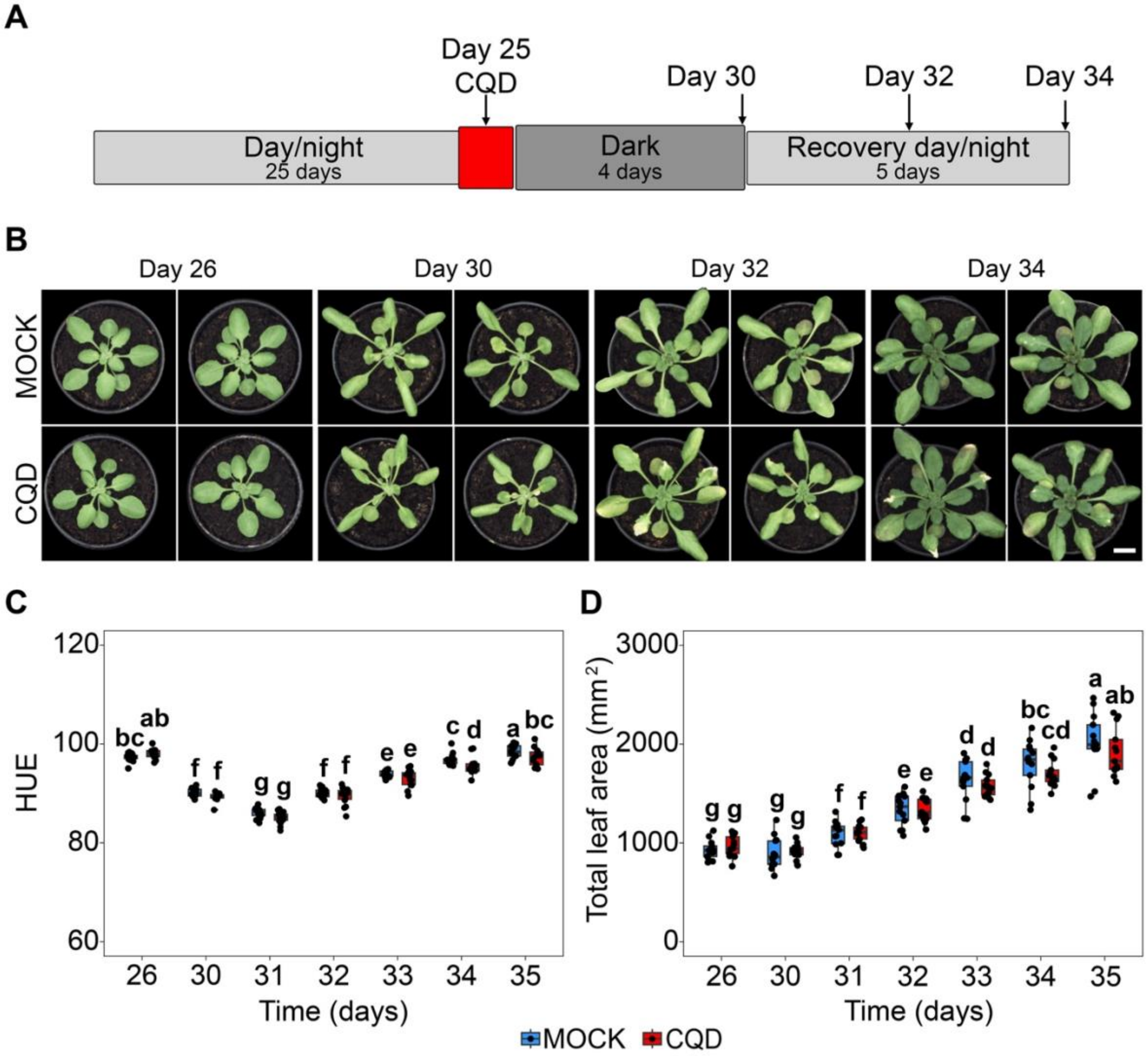

Figure S11. Dark treatment as a control of submergence after CQD application. (A) Schematic representation of the experimental design followed for the control treatment of submergence experiment. Plants were grown under aerobic conditions for 25 days and then sprayed once on the leaf surface with either a CQD or MOCK solution at the end of the day (ZT10, red square). Plants were transferred to continuous darkness 24 hours after MOCK and CQD treatment. (B) Representative pictures of plants before (day 26) and after dark treatment (day 30, 32 and 34) under day/night cycles. Scale bar = 1 cm. (C) HUE index of MOCK- and CQD-treated plants before (day 26) and after (day 30-34) dark treatment (n=13). (D) Quantification of total leaf area before and after dark treatment (n=13). Kruskal-Wallis test followed by post-hoc Wilcoxon

test with multiple testing correction (Benjamini-Hochberg) was carried out in (C) and (D) to calculate groups with significant differences (p-value < 0.05), which are indicated with different letters.

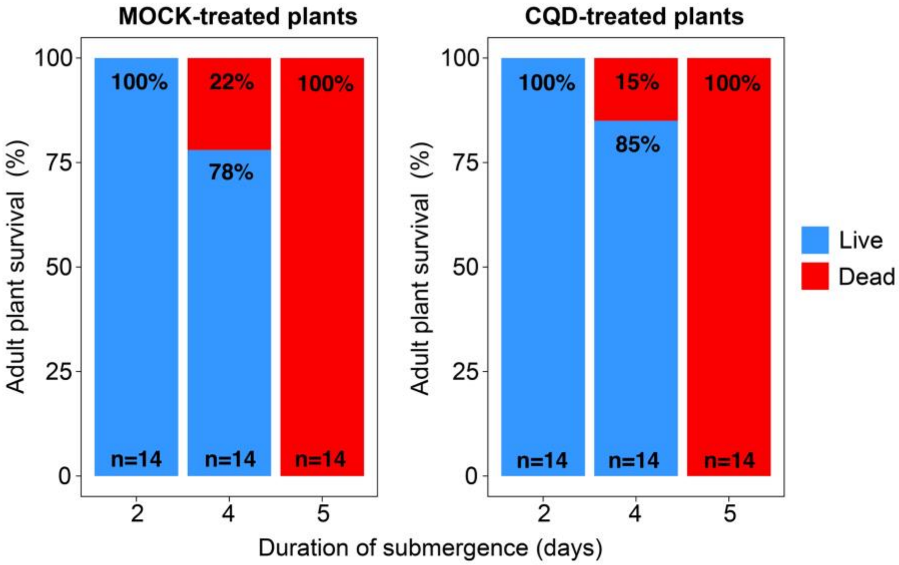

Figure S12. CQD treatment did not improve plant survival across different submergence durations. Survival rate of plants scored after 1 week following 2, 4 and 5 days of submergence, expressed as a percentage of the total sample (n = 14). Plants were grown under aerobic conditions for 25 days and then sprayed once on the leaf surface with either a CQD (100 μM) or MOCK solution at the end of the day (ZT10). After 24 hours, plants were submerged for 2, 4 or 5 days. The percentage of plant survival after 4 days of submergence was extrapolated from Figure 6.

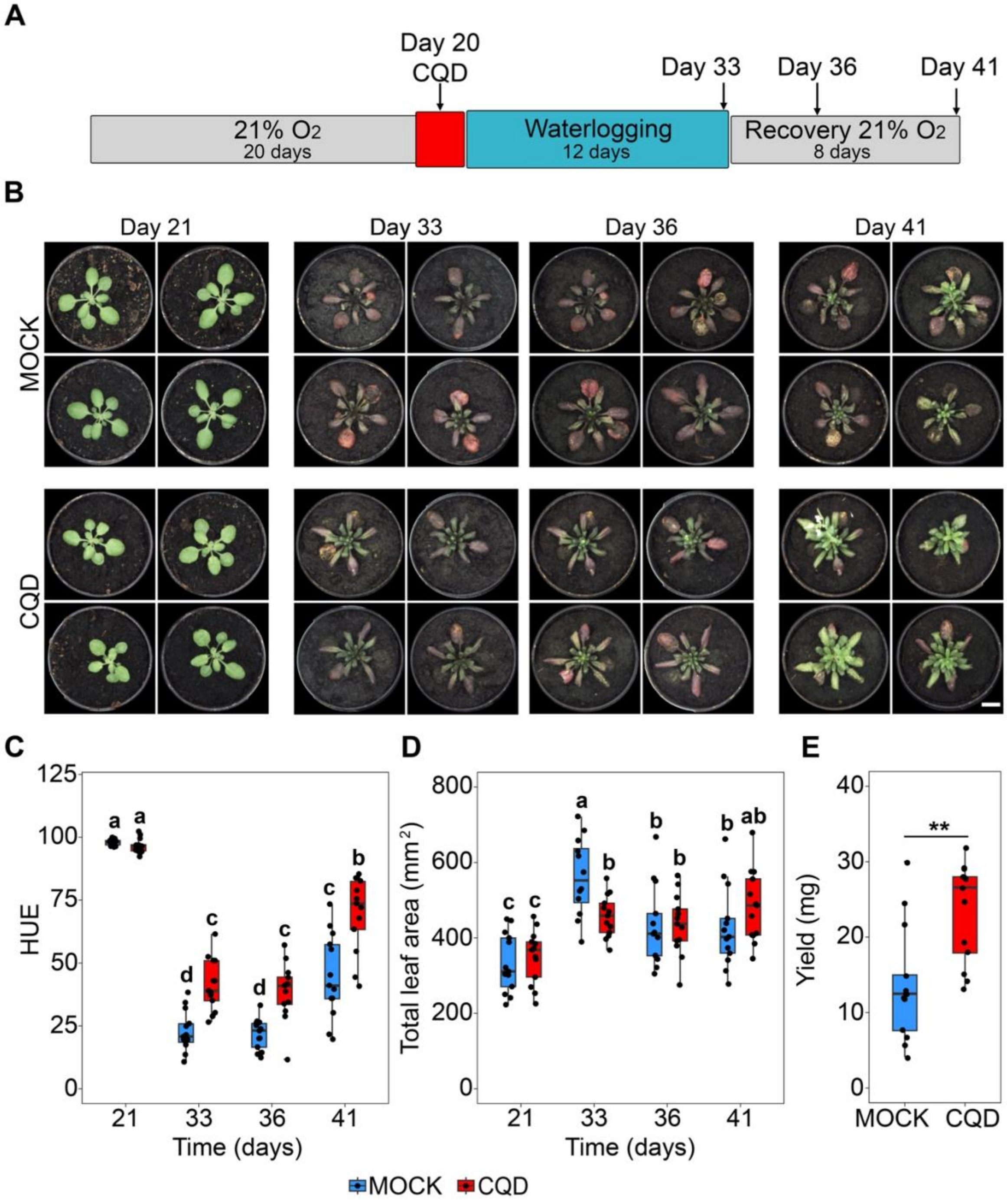

Figure S13. CQD treatment enhances plant tolerance to waterlogging. (A) Schematic representation of the experimental design followed to test the priming effect induced by CQD pre-treatment. Plants were grown under aerobic conditions for 20 days and then once on the leaf surface with either a CQD or MOCK solution at the end of the day (ZT10; red square). Waterlogging treatments were started on the day after treatment with plants having all the root

system under water, but with the petioles and leaves above the water level. (B) Representative pictures of plants grown under waterlogging conditions for 12 days and during recovery phase under aerobic conditions. Scale bar = 1 cm. (C) HUE index of MOCK and CQD treated plants before and after waterlogging at the indicated time points (n=13). (D) Quantification of total leaf area before and after waterlogging (n=13). In (C) and (D) Kruskal-Wallis test followed by post-hoc Wilcoxon test with multiple testing correction (Benjamini-Hochberg) was carried out to calculate groups with significant differences (p-value < 0.05), which are indicated with different letters. (E) Seed production (total harvested seeds per plant) from MOCK and CQD treated plants. *t*-test, *p-value < 0.05, **p-value < 0.01, ***p-value < 0.001.

Table S1. List of bioactive molecules used for the high-throughput chemical genetic screen.
Table S2. Quantification of *pADH::LUC* reporter activity for 2237 bioactive molecules.
Table S3. List of potential chemical inducers found from the initial the high-throughput chemical genetic screen and selected for validation.
Table S4. List of primers used in this study.
Movie S1. Timelapse of *p5xHRPE::LUC* reporter activity from 3-week-old Arabidopsis plants after MOCK or CQD treatment for 24 hours.
